# Lifshitz transition enabling superconducting dome around a charge-order critical point

**DOI:** 10.1126/sciadv.adl3921

**Published:** 2024-07-05

**Authors:** Roemer D. H. Hinlopen, Owen N. Moulding, William R. Broad, Jonathan Buhot, Femke Bangma, Alix McCollam, Jake Ayres, Charles J. Sayers, Enrico Da Como, Felix Flicker, Jasper van Wezel, Sven Friedemann

**Affiliations:** ^1^ HH Wills Physics Laboratory, University of Bristol, Tyndall Avenue, Bristol BS8 1TL, UK.; ^2^ Institut Néel CNRS/UGA UPR2940, 25 Avenue des Martyrs, Grenoble 38042, France.; ^3^ High Field Magnet Laboratory (HFML-EMFL), Radboud University, Toernooiveld 7, Nijmegen 6525 ED, Netherlands.; ^4^ School of Physics, University College Cork, Cork, Ireland.; ^5^ Department of Physics, University of Bath, Bath BA2 7AY, UK.; ^6^ School of Physics and Astronomy, Queen’s Buildings North Building, 5 The Parade, Newport Road, Cardiff CF24 3AA, UK.; ^7^ Institute for Theoretical Physics, University of Amsterdam, Science Park 904, Amsterdam, 1098 XH, Netherlands.

## Abstract

Superconductivity often emerges as a dome around a quantum critical point (QCP) where long-range order is suppressed to zero temperature, mostly in magnetically ordered materials. However, the emergence of superconductivity at charge-order QCPs remains shrouded in mystery, despite its relevance to high-temperature superconductors and other exotic phases of matter. Here, we present resistance measurements proving that a dome of superconductivity surrounds the putative charge-density-wave QCP in pristine samples of titanium diselenide tuned with hydrostatic pressure. In addition, our quantum oscillation measurements combined with electronic structure calculations show that superconductivity sets in precisely when large electron and hole pockets suddenly appear through an abrupt change of the Fermi surface topology, also known as a Lifshitz transition. Combined with the known repulsive interaction, this suggests that unconventional *s*_±_ superconductivity is mediated by charge-density-wave fluctuations in titanium diselenide. These results highlight the importance of the electronic ground state and charge fluctuations in enabling unconventional superconductivity.

## Teaser

Superconductivity emerges once electronic reconstruction releases electrons coupled by charge order.

## INTRODUCTION

Density waves (DWs) underlie the magnetic and charge order in many materials, with well-known examples including CePd_2_Si_2_, BaFe_2_As_2_, and TiSe_2_ (*1*–*3*). DWs are usually formed by a periodic spatial variation of charge [charge density wave (CDW)] or spin [spin density wave (SDW)] at a characteristic wave vector $\vec{Q}$ (*4*). Continuous DW phase transitions are accompanied by diverging fluctuations. In the vicinity of a quantum critical point (QCP) (where the transition is suppressed to zero temperature), the critical fluctuations at $\vec{Q}$ associated with the suppressed DW order can give rise to non-Fermi liquid behavior and are strong contenders for mediating superconductivity in many unconventional superconductors including iron pnictides, heavy-fermion materials, and possibly cuprate high-temperature superconductors (*5*–*7*). For instance, in iron pnictides, SDW fluctuations promote unconventional *s*_±_ superconductivity by coupling electron and hole pockets connected by $\vec{Q}$ (*5*). Although, theoretically, no distinction is made between spin and charge fluctuations in QCP phenomenology, in practice, superconductors linked to soft CDW fluctuations are distinctly lacking.

A dome of superconductivity around a QCP is taken as one of the hallmarks of unconventional superconductivity. Prominent SDW systems demonstrate such a dome, including the heavy-fermion compound CePd_2_Si_2_ (*1*), iron-pnictide BaFe_2_As_2_ (*2*), and the organic superconductor (TMTSF)_2_PF_6_ (*8*). By contrast, a competition of DW order and superconductivity is expected in the scenarios where both orders compete for the same electronic states. Such a competition appears to be realized in prototypical NbSe_2_ (*9*–*11*), where *T*_CDW_ and *T*_c_ are anticorrelated when studied under hydrostatic conditions on strain-free samples (*11*). Complex interaction between CDW order and superconductivity has been observed in underdoped (*12*) and overdoped (*13*) cuprates and nickelates (*14*). Multiple domes inside and outside the CDW phase have been observed in frustrated and topologically nontrivial AV_3_Sb_5_ (A = K, Rb, and Cs) with the possibility of time-reversal symmetry breaking (*15*–*17*).

The transition metal dichalcogenide TiSe_2_ hosts a prototypical CDW associated with a doubling of the lattice constants in all directions $\left[\vec{Q}=\left(\frac{1}{2},\frac{1}{2},\frac{1}{2}\right)\right]$ and a transition temperature of *T*_CDW_ = 202 K (*18*). The CDW gaps out states from electron bands at *L* and hole bands at Γ that are mapped (“nested”) onto each other by $\vec{Q}$. Evidence exists for both electron-phonon and excitonic contributions to the CDW mechanism (*19*–*23*), and the CDW may be chiral (*24*). Previous studies found that the CDW is suppressed and superconductivity can be induced by Cu intercalation (*3*), high pressure (*25*, *26*), or gating (*27*). Combined with its structural simplicity and lack of magnetic order, TiSe_2_ thus provides an ideal setting to investigate the emergence of superconductivity around a putative CDW QCP. Under hydrostatic conditions, the CDW transition is continuously suppressed to zero temperature at a pressure of *P*_CDW_ = 5.1(2) GPa, as observed by x-ray diffraction and magnetotransport measurements (*26*, *28*). Hence, this suggests a CDW QCP at *P*_CDW_ with divergent fluctuations at $\vec{Q}$ . Critical fluctuations have been observed directly with x-ray scattering at the ambient pressure CDW transition at *T*_CDW_, i.e., in the classical limit (*29*). These fluctuations are expected to persist around the CDW transition even when it is suppressed to zero temperature at *P*_CDW_ although a direct measurement of quantum critical fluctuations in TiSe_2_ remains elusive (*26*). Hence, while the continuous suppression of *T*_CDW_ satisfies the definition of a CDW QCP, it will require further detailed studies to probe whether quantum fluctuations exist at the QCP. Nevertheless, a minimum in the quasiparticle lifetimes and modifications of the resistance power law suggest the presence of soft modes and/or fluctuations at *T*_CDW_ and *P*_CDW_, respectively (*25*, *28*, *30*). However, the link between superconductivity and CDW order in TiSe_2_ remains elusive, as the exact location of superconductivity relative to the CDW QCP remains unclear (*3*, *25*, *26*, *28*, *31*–*34*). We report that TiSe_2_ manifests a dome of superconductivity centered around the CDW QCP under improved hydrostatic pressure conditions.

In addition, we investigate the interplay between CDW order, superconductivity, and the electronic ground state in 1*T*-TiSe_2_. Of particular interest is the topology of the Fermi surface, which, in some cases, can change abruptly in a so-called Lifshitz transition (*35*). Lifshitz transitions can stabilize new phenomena (*36*–*39*), including superconductivity (*40*–*43*). Identifying these Lifshitz transitions offers a glimpse of the mechanism that mediates these ordered states. However, identifying Lifshitz transitions remains challenging because of the difficulty to accurately determine the electronic ground state.

We study the intrinsic behavior of pristine samples using hydrostatic pressure to fully suppress the CDW phase of TiSe_2_. We measure the low-temperature resistance to map the superconducting dome, observe quantum oscillations across the full CDW phase and beyond, and combine these results with density functional theory (DFT) and a tight-binding analysis to map the evolution of the electronic structure identifying two Lifshitz transitions. We find that the onset of superconductivity coincides with the emergence of a major hole and electron pocket at one of the Lifshitz transitions. Combined with earlier theoretical work (*44*), this suggests that TiSe_2_ hosts unconventional superconductivity with *s*_±_ interband pairing at the CDW $\vec{Q}$ vector.

## RESULTS

### Superconductivity at the CDW QCP in TiSe_2_

**Fig. 1.**
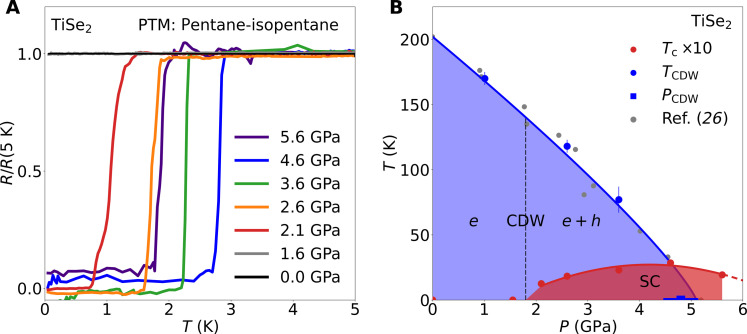
Superconductivity around the CDW QCP in TiSe_2_. (**A**) Resistance measurements using a hydrostatic PTM demonstrate superconductivity over an extended pressure range in TiSe_2_. (**B**) Superconducting transition temperatures *T*_c_ defined as the temperature at which the resistance is reduced to 90% of the normal-state resistance. The dashed vertical line indicates the pressure at which we observe a Lifshitz transition. *T*_CDW_ (*P*) extracted from resistance measurements (see section S1) and *P*_CDW_ extracted from the jump of quantum oscillation frequencies (see Fig. 3) are included in (B) (blue circles and squares, respectively). These data agree with previous high-pressure x-ray diffraction measurements (gray points) performed using a similarly high-quality pressure medium (*26*, *28*). Solid lines and shading are guides to the eye.

We observe that the superconducting transition temperature *T*_c_ forms a dome around the CDW QCP. Our resistance measurements on pristine TiSe_2_ tuned with hydrostatic pressure are shown in Fig. 1. While TiSe_2_ remains a normal metal at ambient and low pressures down to at least 60 mK, we find sharp superconducting transitions at pressures P > 2.0 GPa. The resistance transition is suppressed in magnetic field as expected for a superconductor (see section S1). Previously, superconductivity was observed between 2.0 and 3.5 GPa with a maximum transition temperature $T_{c}^{\text{max}}=1.8\;K$ in a study using a solid pressure medium (*25*). By using the liquid pressure transmitting medium (PTM) 1:1 pentane-isopentane, we improve on the hydrostatic conditions. We find that superconductivity still sets in at 2 GPa but now extends to at least 5.6 GPa, with an enhanced maximum transition temperature $T_{c}^{\text{max}}=2.9\;K$ close to the CDW QCP. The presence of superconductivity beyond *P*_CDW_ rules out the earlier suggestion (*26*) that superconductivity is confined to the domain walls of the CDW and requires a new understanding.

The comparison with previously published results underlines the sensitivity of both superconductivity and CDW order to pressure conditions. Our study shows a 50% higher $T_{c}^{\text{max}}$ under hydrostatic conditions than previous studies using solid pressure media (*25*, *32*, *33*). This sensitivity of the superconductivity to nonhydrostatic conditions hints at an unconventional mechanism, possibly with sign change of the superconducting gap. A similar sensitivity to pressure conditions has been summarized for the CDW order: Under the best hydrostatic conditions, the critical pressure of the CDW order is enhanced by more than 50% and reaches *P*_CDW_ = 5 GPa (*26*, *28*).

The observed dome of superconductivity around the CDW QCP in TiSe_2_ raises the question: To what extent does the vicinity of the CDW QCP influence its superconducting properties? Superconductivity is conventionally mediated by phonons, while in many unconventional superconductors, magnetic or nematic fluctuations are suggested to contribute to or even dominate the binding of Cooper pairs (*6*). In heavy-fermion and iron-pnictide superconductors, these magnetic and nematic fluctuations are believed to arise from a QCP (*5*). In TiSe_2_, the fluctuations around the CDW QCP involve both the lattice and the electrons, and possibly excitons (*19*–*22*). The vicinity of the QCP and its associated fluctuations are known to influence the electrons, as manifested in TiSe_2_ in the normal-state resistivity ρ = ρ_0_ + *AT^n^*, which has a dip in the exponent *n* in the vicinity of the QCP (*28*). The value *n* = 3 away from the QCP is consistent with interband scattering driven by phonons (*45*). The reduction to *n* ≈ 2 near the QCP can be interpreted as evidence for an increase of the effective electron-phonon coupling constant λ due to softening of the CDW fluctuations (*46*). A change in disorder, which can also cause a dip in *n*, can be ruled out as the origin for the change in *n*, as we maintain constant chemical purity with pressure and observe quantum oscillations at all pressures studied. By contrast, previous studies using nonhydrostatic conditions and lower purity samples show a reduced dip of *n* and a reduced $T_{c}^{\text{max}}$ , suggesting a smaller λ (*25*, *32*, *33*). In our high-purity samples under hydrostatic pressure, the dip in *n* suggests that the conduction electrons predominantly couple to phonon modes that are softened by the nearby CDW QCP (*30*). Extending this reasoning, the maximum value $T_{c}^{\text{max}}$ of the superconducting transition temperature at the QCP may be similarly enhanced by an increase in λ.

This mechanism of enhancing *T*_c_ through an increase in λ from softened CDW fluctuations is similar to elemental uranium and LuPt_2_In, in which an increase in *T*_c_ correlates with the softening of phonons as observed by inelastic x-ray scattering and specific heat measurements, respectively (*47*, *48*). However, TiSe_2_ hosts a single dome of superconductivity around a CDW QCP, which allows us to study the onset of superconductivity. In particular, we ask, what initiates the superconductivity in TiSe_2_ at 2 GPa? We use quantum oscillation measurements and electronic structure calculations to address this question.

### High-pressure quantum oscillation measurements

**Fig. 2.**
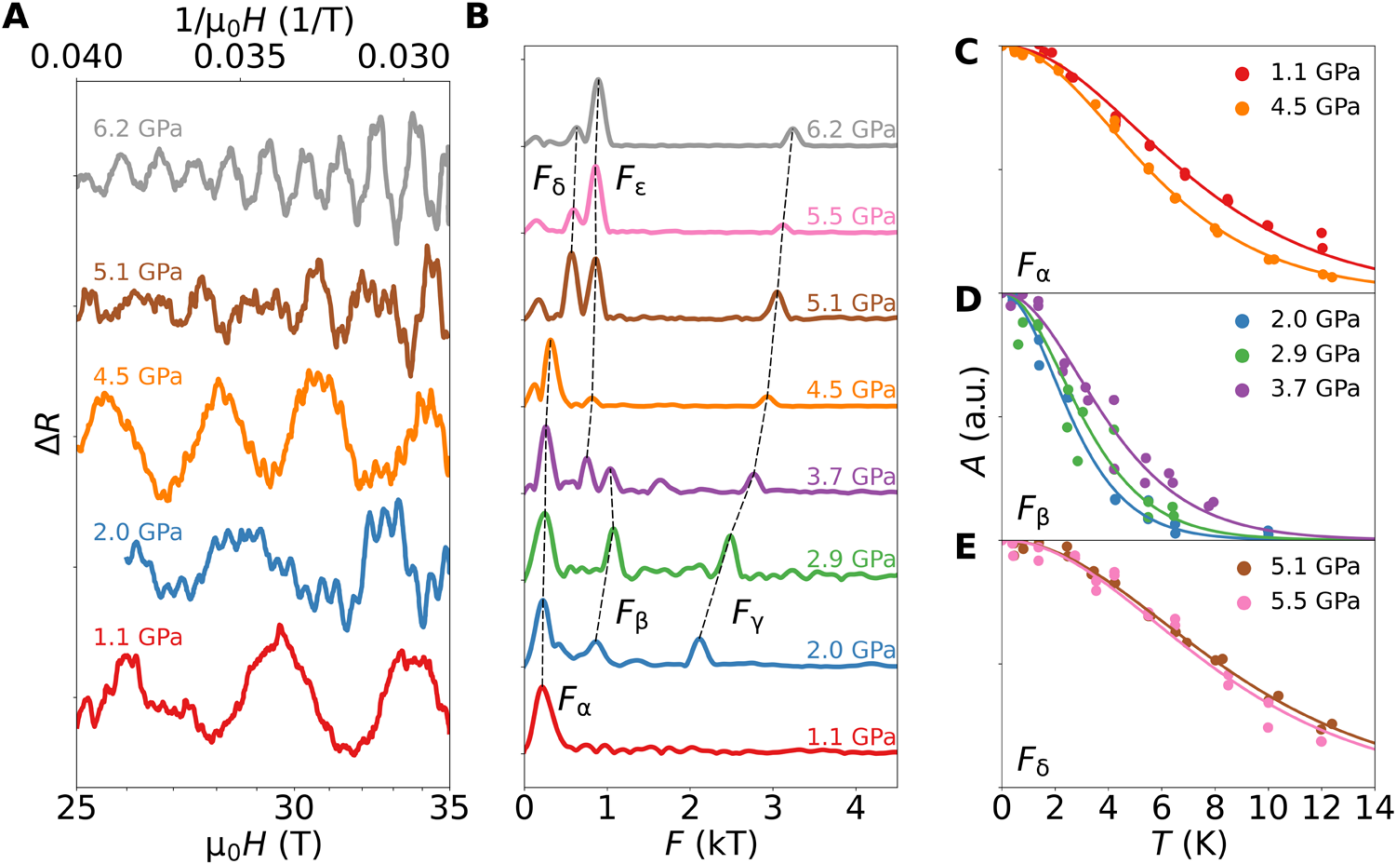
Quantum oscillations in TiSe_2_ at high pressures. (**A**) Resistance after subtraction of a polynomial background (cf. section S2) plotted versus inverse magnetic field (top axis) across a wide range of pressure. (**B**) Fourier transformation of the data gives the oscillation amplitude as a function of frequency. Dashed lines trace quantum oscillation frequencies across pressure. Presented data were measured between 0.32 and 0.46 K except for the 1.1-GPa curve, which was obtained at 1.4 K. (**C to E**) The temperature dependence of the amplitudes for selected frequencies and pressures. Solid lines represent Lifshitz-Kosevich fits used to extract effective masses. a.u., arbitrary units.

The presence of quantum oscillations presented in Fig. 2 demonstrate that we retain the high purity of our samples under hydrostatic pressure tuning (*30*). Analyzing the quantum oscillation measurements affords us the highest resolution and most reliable method to obtain information about the Fermi surface and electronic structure. Previous quantum oscillation measurements have been decisive for studies of electronic structure and Fermi surface reconstruction as well as the quasiparticle renormalization at quantum phase transitions in a range of materials (*49*–*51*).

**Fig. 3.**
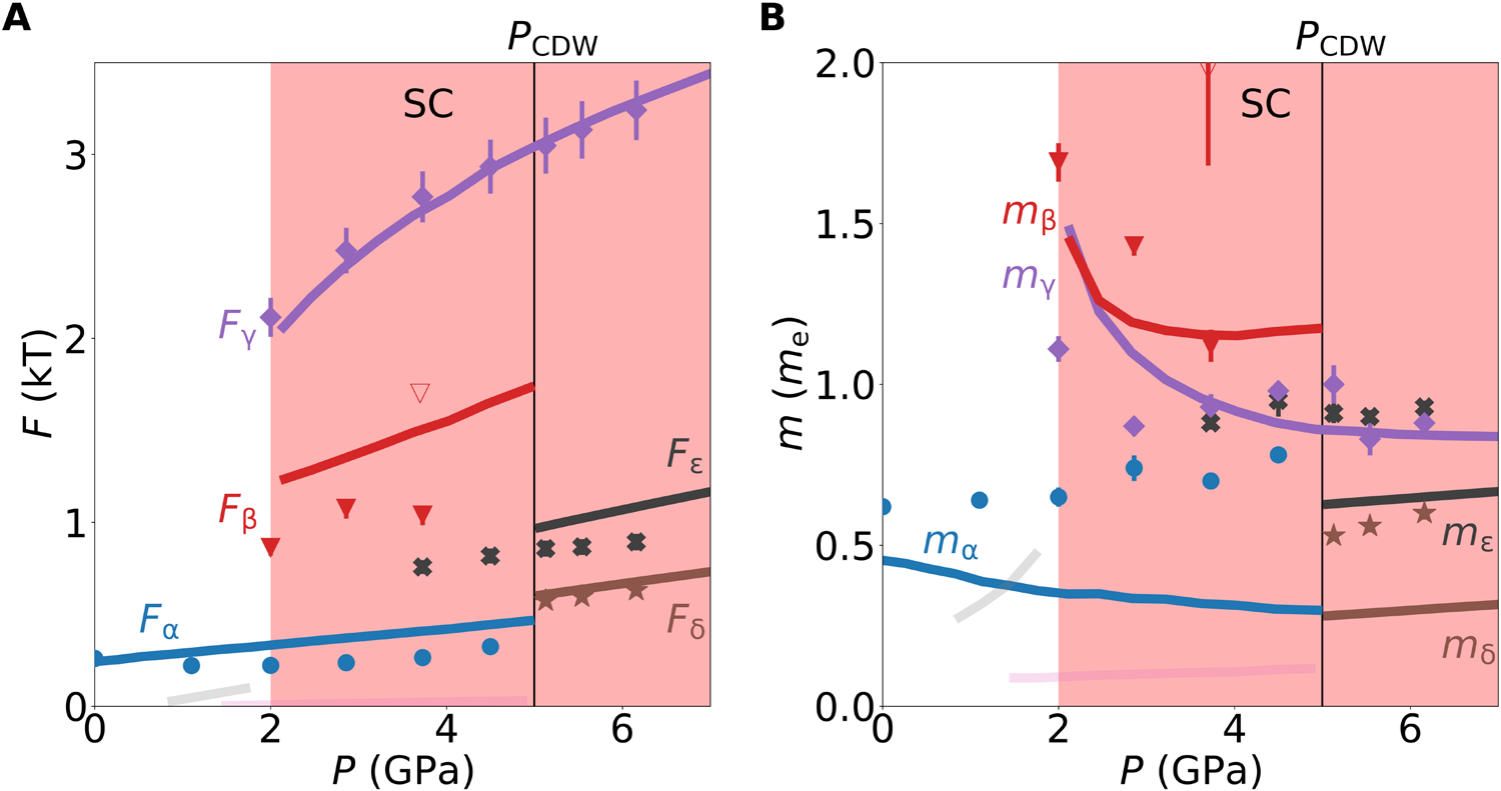
Comparison between modeled and measured quantum oscillations. (**A**) Quantum oscillation frequencies from experiment (symbols) and band-structure calculations (lines). Labels refer to frequencies identified Fig. 2, with subscripts corresponding to orbits in Fig. 4. Faint lines correspond to small frequencies that are not observed experimentally. Neck orbits of the Se band at *k*_z_ = π/*c* are omitted as they are not observed experimentally. Red background indicates the range of superconductivity observed. Open triangle marks a frequency that may result from magnetic breakdown, as discussed in section S3. (**B**) Corresponding effective masses as a function of pressure.

At ambient pressure and low temperature, TiSe_2_ is fully described by a single-electron pocket, which manifests as a single quantum oscillation frequency *F*_α_ = 0.26 kT for magnetic fields along the *c* axis (*30*). This pocket is the result of the Fermi surface reconstruction inside the CDW phase and was previously observed using angle-resolved photoemission spectroscopy (ARPES) (*52*). In particular, the agreement of quantum oscillations with heat capacity data establishes the fact that *F*_α_ corresponds to the only Fermi surface pocket present at ambient pressure (*53*). We observe no other orbits up to 2 GPa, suggesting that the Fermi surface remains restricted to one pocket α. Within this pressure range, *F*_α_ decreases slightly, and the mass evolves smoothly (see Figs. 2 and 3). The sudden emergence of new frequencies at higher pressures provides evidence for two Fermi surface reconstructions taking place at 2 and 5 GPa, respectively (see Fig. 3).

A first Fermi surface reconstruction is identified at 2 GPa where we observe two new frequencies *F*_β_ ≈ 1 kT and *F*_γ_ ≈ 2 kT (see Fig. 2). These are one order of magnitude larger than *F*_α_ and prove the emergence of at least one large new Fermi surface (we remind that heat capacity measurements rule out the presence of such a large pocket at ambient pressure). The emergence of such a large Fermi surface is unexpected, as it happens well before the critical pressure at which CDW order disappears.

A second Fermi surface reconstruction is observed at *P*_CDW_ = 4.8(3) GPa. Here, *F*_α_ ceases to exist, and a new frequency *F*_δ_ ≈ 0.55 kT emerges. *F*_α_ and *F*_δ_ are clearly distinguished by the difference in mass, establishing these as orbits on different Fermi surface pockets. By contrast, *F*_γ_ evolves smoothly through *P*_CDW_ and continues to increase up to the highest pressure of our study (6.2 GPa). Below, our electronic structure calculations model the complex evolution of the Fermi surfaces detected by quantum oscillations, including the two Fermi surface reconstructions.

### Electronic structure calculations

We start our modeling of the observed quantum oscillation frequencies using DFT calculations in WIEN2k (*54*) including spin-orbit coupling and based on the well-established crystal structure of TiSe_2_ without the CDW (space group $P\bar{3}m1$ ). The pressure dependence of the lattice parameters is calculated with DFT and matches the experimental results (see Methods and section S4). The DFT band structure is then used to unambiguously assign the quantum oscillation frequencies above *P*_CDW_ (see Fig. 3) and to calibrate band shift corrections of the electronic structure in the absence of CDW order as detailed in section S4.

**Fig. 4.**
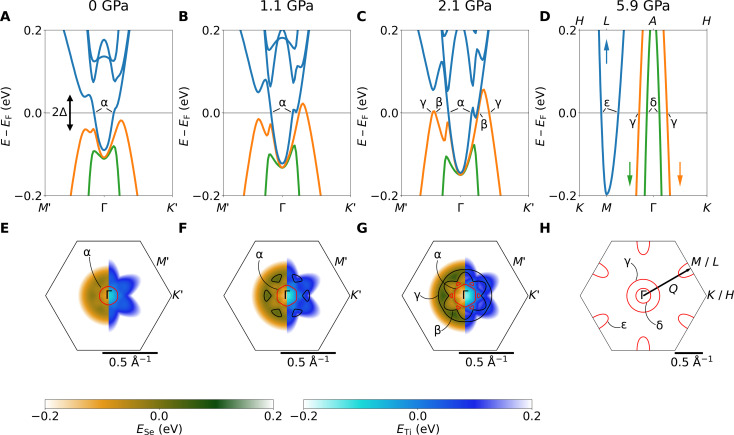
Pressure evolution of the Fermi surface in TiSe_2_. (**A** to **D**) Simulated band structure inside [(A) to (C)] and outside (D) the CDW phase. Blue lines indicate tight binding fits to the Ti 3d electron band back-folded from the *L* point, and green and orange lines indicate the Se 4p hole bands. (**E** to **H**) Corresponding Fermi surface contours in the tight-binding model. The color maps in the left half of each Brillouin zone indicate the energies of the Se tight-binding band with red lines marking the corresponding Fermi surface. The right half of each Brillouin zone with black lines correspond to the Ti bands. Orbits observed in experiment are labeled with Greek letters. (H) Normal-state Fermi surface outside the CDW regime, with one of the three CDW wave vectors $\vec{Q}=\left(\frac{1}{2},\frac{1}{2},\frac{1}{2}\right)$ indicated by the black arrow. We label points on the high-pressure Brillouin zone with Γ, *M*, *L*, *K*, and *H* where *L* is mapped onto *M* and *H* onto *K* in our 2D model. Points in the reconstructed Brillouin zone inside the CDW phase are labeled with Γ, *M*′, and *K*′.

Outside the CDW phase (*P* ≥ *P*_CDW_), DFT calculations provide an excellent match to the experimental frequencies. The three experimental frequencies *F*_γ_, *F*_δ_, and *F*_ε_ are reproduced in both magnitude and pressure dependence. On this basis, we can clearly identify that *F*_γ_ and *F*_δ_ correspond to the outer and inner hole pockets at Γ, respectively, while *F*_ε_ corresponds to the electron pocket at *L* (see Fig. 4). The calibration of band shifts in the DFT calculations (see Methods and section S4) to these observed frequencies provides us with a reliable basis to model the effect of the CDW using a tight-binding approach.

### Pressure dependence of electronic structure

Inside the CDW phase (*P* ≤ *P*_CDW_), we model the Fermi surface using a two-dimensional (2D) tight-binding model with band parameters extracted from our calibrated DFT calculations. We include a simple *k*-independent orbitally nonselective CDW gap Δ between the Se and Ti bands (see Methods). The gap size Δ(*P*) is taken proportional to *T*_CDW_(*P*) at all pressures, with an estimate of Δ(0) = 40 meV at ambient pressure based on TiSe_2_ being a moderately strong coupling CDW with a transition temperature of *T*_CDW_ = 202 K (see Methods). Our model quantitatively accounts for the observed quantum oscillation frequencies over the full pressure range studied, as shown in Fig. 3.

The tight-binding model naturally reproduces the Fermi surface reconstruction at *P*_CDW_ = 5 GPa identified by the change from *F*_δ_ outside the CDW to *F*_α_ inside the CDW phase, as shown in Fig. 3. The tight binding model also predicts the disappearance of *F*_ε_ and emergence of *F*_β_ when entering the CDW phase from high pressures. In experiment, we observe *F*_ε_ to continue below *P*_CDW_, and *F*_β_ is only detected below 4 GPa. The fact that *F*_β_ is not observed in experiment right up to *P*_CDW_ may be due to the low intensity of this frequency as a result of either geometrical factors or reduced quasiparticle weight. The continued observation of *F*_ε_ into the CDW phase and the additional frequency observed at 3.7 GPa are possibly results of magnetic breakdown as detailed in section S3 (*55*). The effective masses *m*^⋆^ measured for *F*_δ_ and *F*_ε_ are enhanced compared to the calculated band mass by ≈80% for *F*_δ_ and *F*_ε_ (cf. black and purple symbols compared to same color lines in Fig. 3B).

The new orbits *F*_α_ and *F*_β_ inside the CDW phase are identified as the inner and outer closed electron orbits around Γ arising from the hybridization between the three elliptical electron bands originally at *L* and the smaller hole band originally at Γ. In our model, the α pocket persists down to ambient pressure, where it encompasses the entire Fermi surface in excellent agreement with experiments (*30*). We assign the difference between observed and calculated frequencies for *F*_α_ to the limitations of our model. Quantitative prediction of small frequencies is usually most susceptible to details of the model. However, our main findings below are based on the pressure evolution of the large orbits, e.g., *F*_γ_. The comparison of the measured and calculated masses reveals an enhancement of *m*^⋆^ for *F*_α_ rising from ≈20% to ≈200% on approaching *P*_CDW_. This enhancement is consistent with an increasing electron-phonon coupling on the approach of *P*_CDW_.

Small differences between the calculated and observed frequency *F*_ε_ are a result of the limited expansion of the used tight-binding model. By extension, while the calculated *F*_β_ is seen to be overestimated in Fig. 3, we show in section S5 that the DFT obtains this frequency correctly and the difference is entirely due to the limited expansion in our tight-binding model. This limitation does not affect our analysis of the effective mass. The measured effective mass follows the trend obtained from the tight-binding calculations with a strong enhancement toward the disappearance of *F*_β_ upon lowering the pressure down to 2 GPa.

The tight-binding model reproduces the continuity of the highest frequency, *F*_γ_, deep into the CDW phase and matches the evolution of its effective mass. The large circular hole pocket associated with *F*_γ_ does not hybridize with the back-folded elliptical electron pockets immediately upon entering the CDW (see Fig. 4H). Instead, the electron bands hybridize with the small hole band and form a six-leaf flower-shaped electron pocket (see Fig. 4G). Only at *P* ≤ 2 GPa do the electron pockets ε hybridize with the hole band γ because only below this pressure does the CDW gap overcome the energy difference and separation in *k*-space between γ and ε (see Fig. 4, F and G). The hybridization below *P* ≈ 2 GPa drives the disappearance of *F*_β_ and *F*_γ_ and marks a Lifshitz transition. As a result, most of the carriers are gapped away from the Fermi level below 2 GPa (high-quality nesting), whereas a minority of carriers are gapped above 2 GPa (poor-quality nesting), while the semimetallic overlap increases substantially with increasing pressure, in agreement with the large decrease in residual resistivity (*28*). Thus, our tight-binding model confirms and identifies the experimental Lifshitz transition inside the CDW at 2 GPa.

The onset of superconductivity at 2 GPa is observed to coincide exactly with the emergence of large electron and hole pockets around Γ. These pockets originate from *L* and Γ, respectively, and are connected by the CDW wave vector $\vec{Q}$ in the unreconstructed Brillouin zone. The abrupt emergence of superconductivity at this Lifshitz transition therefore suggests a close relationship between the superconducting and CDW order, as well as an interband character for the Cooper pairs. This tangible possibility for the emergence of interband superconductivity in TiSe_2_ under pressure raises the question whether the same mechanism may also be at play in Cu-intercalated TiSe_2_.

### Doping dependence of electronic structure

**Fig. 5.**
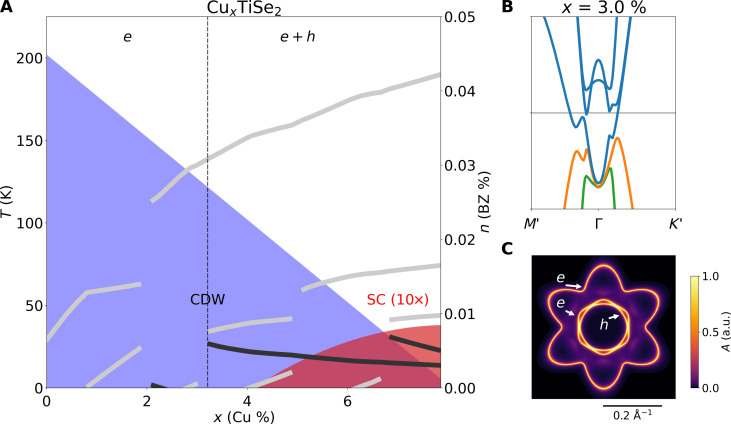
Doping evolution of the Fermi surface in Cu*_x_*TiSe_2_. (**A**) Calculated evolution of the carrier concentration (right axis) for the electron (gray) and hole (black) Fermi surface pockets, as a function of Cu intercalation *x*. Blue and red shaded areas mark the experimental transition temperatures (left axis) of the CDW and superconductivity, respectively, as reported in (*3*). BZ, Brillouin zone. (**B**) Electronic structure and Fermi surface topology illustrating the emergence of a hole pocket when crossing the Lifshitz transition at *x* ≈ 3%. Blue lines indicate the Ti 3d electron bands back-folded from the *L* points, while green and orange lines mark the Se 4p hole bands. (**C**) Total spectral weight at the Fermi level showing the reconstructed Fermi pockets at the same doping as (B). The full pockets are visible as a result of finite broadening of the bands for the illustration of spectral weight.

To model the Fermi surface evolution of Cu-intercalated Cu_x_TiSe_2_, we investigate the doping dependence in our tight-binding model and compare with experimental results of (*3*). In our tight-binding model, the ambient pressure semimetallic electronic structure is established from the match to quantum oscillation measurements (see Methods for details of the semimetallic ground state). The Cu intercalation is modeled by raising the chemical potential to reflect an electron doping of 0.45 electrons per Cu, as determined from supercell DFT (*56*) and consistent with Hall effect and ARPES measurements (*57*–*59*). The doping leads to the growth of the electron pockets originating from the *L* point (compare Fig. 5B with Fig. 4A). Combined with the suppression of the CDW gap Δ(*x*) [scaled to the experimental *T*_CDW_(*x*) (*3*, *31*, *60*) as before], this leads to the occurrence of two similar Lifshitz transition as observed under pressure. The first takes place well inside the CDW phase, while the second is linked to the destruction of the CDW order at *x* ≈ 8%.

The first Lifshitz transition is predicted for Cu intercalation at *x* ≈ 3%, in close proximity to the experimentally observed onset of superconductivity at *x* ≈ 4% (*3*). In Fig. 5, we plot the carrier concentrations for individual electron and hole bands (gray and black lines). The electron concentration grows with Cu intercalation as expected. At lowFor *x* < 3%, only a single-electron pocket is present because the CDW gap is large and the separation of the underlying electron pockets from *L* and hole pockets from Γ is small—gapping out all hole bands. Above *x* ≈ 3%, the CDW gap becomes too small to overcome the growing separation and a hole pocket emerges marking a Lifshitz transition (see Fig. 5). The proximity of the Lifshitz transition with the onset of superconductivity suggests that superconductivity in Cu_x_TiSe_2_ is dependent on the presence of both electron and hole bands and thus also of interband character.

## DISCUSSION

We have established that superconductivity exists in a dome centered around the pressure-induced CDW QCP in TiSe_2_ with $T_{c}^{\text{max}}=2.9\;K$ . This observation unifies the pressure and doping phase diagram (*3*) and suggests that superconductivity is mediated by the CDW fluctuations that soften on approach of the CDW QCP under both tuning parameters. Soft fluctuations near a QCP can manifest as an enhancement and divergence of the effective masses of electrons. In TiSe_2_, we observe a pressure-independent (i.e., nondivergent) mass enhancement of about a factor of 2 over the DFT masses for *F*_δ_ and *F*_ε_, testifying the presence of interactions across the phase diagram.

For one frequency, *F*_α_, we observe the mass enhancement increasing gradually toward the CDW QCP up to a factor of 3 over the tight-binding model (see Fig. 3), providing evidence for a moderate enhancement of interactions at the CDW QCP. Combined with the evidence for a peak in electron-phonon coupling deduced from the minimum in the exponent of the temperature dependence of the resistivity (*28*), this indicates that the CDW fluctuations play a role in the peak of the superconductivity. These fluctuations may involve phonons, electrons, excitons, or a combination. This highlights that the peak of the superconductivity is related to the nearby CDW QCP similar to other quantum critical superconductors.

In addition, for both high-pressure and Cu-intercalated TiSe_2_, we have established the coincidence of a Lifshitz transition with the onset of superconductivity. In both cases, we conclude that the presence of electron and hole pockets originating from *L* and Γ, respectively, are a prerequisite for superconductivity, suggesting that the superconductivity is of interband nature, with pairing enabled by the presence of a CDW with wave vector $\vec{Q}$ . A previous renormalization group study found that interband pairing mechanisms are repulsive in TiSe_2_ (*44*). This implies that the interband superconductivity in TiSe_2_ may be of unconventional *s*_±_ type in which the superconducting order parameter changes sign between the electron and hole pockets. If upheld, then the emergence of superconductivity in TiSe_2_ closely parallels the established mechanism for interband *s*_±_ superconductivity in the iron pnictides, with the difference that *s*_±_ pairing is promoted by a CDW.

These results are relevant well beyond the study of TiSe_2_. For instance, a Lifshitz transition has been observed in Ba(Fe_1−*x*_Co*_x_*)_2_As_2_ in which electron and hole pockets connected by the SDW *Q* vector partially emerge at the Fermi level well before the end of the SDW phase at the onset of *s*_±_ superconductivity (*40*), closely resembling the situation discussed here. Likewise, superconductivity in magic-angle trilayer graphene is known to be bounded by Lifshitz transitions (*43*). In quasi-1D Bechgaard salts, superconductivity is similarly driven by increasingly mismatched nesting under pressure (*61*, *62*). In the heavy-fermion compound URhGe, the magnetic field–induced superconductivity has been proposed to result from a Lifshitz transition (*41*). Combined, these cases form unexpectedly universal evidence of Lifshitz transitions demarcating the onset of superconductivity. This includes systems with or without divergent effective masses, with a prototypical Fermi liquid dispersion or in the presence of Dirac cones, in quasi-1D, -2D, or -3D and with charge or spin interactions considered dominant in Cooper pair formation. For the specific case of TiSe_2_, charge order, as opposed to spin order, underlies the emergence of a superconducting dome in the phase diagram. Our results suggest that Lifshitz transitions are an overlooked prerequisite for the emergence of unconventional superconductivity at the edge of DW phases in other materials, especially concerning regions of coexistence.

## METHODS

### High-pressure resistance and quantum oscillation measurements

Studies of quantum oscillations and superconductivity were conducted on the very same sample #1 in a moissanite anvil pressure cell (MAC) machined from CuBe. Details about sample growth can be found in section S1.

Moissanites in the MAC had a culet size of 800 μm. Metallic gaskets were prepared by indenting 450-μm-thick BeCu to ~60 μm, followed by drilling a 450-μm hole. These were insulated with a mixture of alumina and 1266 stycast, which was cured at high pressure between the anvils such that the total thickness of the gasket was less than 100 μm. A 400-μm hole was then drilled in the insulation to act as the sample chamber. Six bilayer electrodes were deposited on the anvil by firstly sputtering 20 μm of nichrome, followed by thermally evaporating 150 μm of gold; excess nichrome was removed with TFN (Transene) etchant. Last, gold electrodes were deposited directly onto the sample that were electrically connected to the electrodes on the culet with EE129-4 (Epotek) silver epoxy, which was left to cure at room temperature to avoid degrading the sample.

The pressure medium used was a 1:1 mixture of pentane and isopentane, which is hydrostatic up to 7.4 GPa (*63*). The pressure was determined at room temperature by ruby fluorescence from multiple ruby flakes within the sample chamber. The uncertainty in the pressure reflects the variation of pressure between all the rubies across the sample chamber and before and after cooldown.

Quantum oscillations were measured at the High Field Magnet Laboratory (HFML), Nijmegen, in a helium-3 cryostat with the MAC submerged in helium-3. The maximum magnetic field applied was 35 T, and the field was swept at different rates below 60 mT/s; the slowest rate used was 10 mT/s to resolve the highest frequency oscillations. A four-probe method with SR830 lock-in amplifiers was used to measure the resistance and a Keithley 6221 applied a maximum current of 0.5 mA to avoid heating the sample. Effective masses were determined from Lifshitz-Kosevich fits to the resistance between 25 and 35 T for all pressures and frequencies. Low frequency peaks are discussed in section S2.

Subsequent ^3^He–^4^He dilution refrigerator measurements were performed on depressurization for detailed measurements of the superconductivity. Here, the MAC was mounted in vacuum and coupled to the mixing chamber through a metallic sample holder. The four-point AC resistivity method was operated at around 19 Hz and used model 1900 transformers for signal amplification. To avoid any risk of heating the sample, we test for heating and find no observable shift in *T*_c_ or *H*_c2_ at 20 μA, but out of an abundance of caution, we typically use 5 μA, decreasing to 1 μA at 50 mK. Example *H*_c2_ data are shown in section S1. The residual resistance observed in Fig. 1 in the superconducting state likely results from stray capacitative coupling.

### DFT calculations

We use WIEN2k with spin-orbit coupling in the Perdew-Burke-Ernzerhof (*64*) basis set. First, we calculate the pressure dependence of the lattice parameters. This is done by fitting the equation of state to the evolution of the total energy as a function of unit cell volume. The total energy is obtained by relaxing the internal degrees of freedom for fixed unit cell volume. In particular, we minimize the total energy to find the *z* and *c*/*a* parameters for each unit cell volume. Here, *zc* is the distance of the Se atoms out of the plane. Subsequently, we fit the Birch-Murnaghan equation of state to the evolution of energy versus unit cell volume to convert the volume to pressure. Example fits and a comparison between lattice parameter evolution and x-ray diffraction under pressure are shown in section S4. For details of the doping evolution of the ambient pressure electronic structure, see section S6.

In the absence of a CDW above 5 GPa, we unambiguously assign the quantum oscillations with the DFT results. We use band shifts up to 150 meV to get the best match between DFT and experiment (see section S4 for details). We maintain a slight electron doping throughout, and although the band shifts reduce the semimetallic overlap compared to natural DFT, we obtain semimetallic character at all pressures. These results show that TiSe_2_ is a semimetal at high pressures with band overlap between electron and hole bands at *L* and Γ, respectively. Previous magnetotransport studies show that the zero-pressure parent state (*T* > *T*_CDW_) has both electrons and holes thermally populated. Even in the extrapolated *T* → 0 limit, the non-CDW state retains a finite semimetallic overlap at ambient pressure (*30*). This semimetallic state at ambient pressure is also obtained in our DFT model (see section S4). We note that the quasi-2D neck orbits of the hole bands at the *A* point predicted by DFT are not detected in quantum oscillations, in agreement with ARPES (*52*), specific heat (*53*), and magnetotransport (*30*).

### Implementation of the CDW

To implement the CDW, we fit a tight binding model to the DFT bandstructure as detailed in section S4 (*65*). We restrict ourselves to the *k_z_* = 0 plane at Γ and the *k_z_* = π/*c* plane at *L* within ±150 meV of the Fermi level. Subsequently, we implement a CDW reconstruction using the following Hamiltonian:

$$\hat{H}\left(\vec{k}\right)=\left(\begin{array}{ccccc} E_{\vec{k}}^{4p} & 0 & \Delta & \Delta & \Delta \\ 0 & E_{\vec{k}}^{4p\prime } & \Delta & \Delta & \Delta \\ \Delta & \Delta & E_{\vec{k}-\vec{Q}}^{3d} & 0 & 0\\ \Delta & \Delta & 0 & E_{\vec{k}-R\left(\vec{Q}\right)}^{3d} & 0\\ \Delta & \Delta & 0 & 0 & E_{\vec{k}-R^{2}\left(\vec{Q}\right)}^{3d}) \end{array}\right)$$ (1)

Here,$\vec{Q}$ indicates the vector from Γ to one of the *L* points. *R* denotes rotation under 120° with respect to the *c* axis to cover the three inequivalent *L* points. The CDW gap Δ is approximated as independent of $\vec{k}$ or orbital. This choice was made because no orthogonality exists between out-of-plane antibonding Se 2p*_z_* orbitals and in-plane Ti 3d orbitals, unlike the *p*_*x*,*y*_ orbitals relevant for, e.g., monolayers of the sister compound TiTe_2_ (*66*). We add a small band-independent linear-in-*P* energy shift of ±12 meV/GPa to all bands, keeping the bands unchanged at *P*_CDW_, such that the band overlap reduces at low pressure, which was applied to fit the ambient pressure quantum oscillation frequency.

We now outline our choice for gap size at ambient pressure. Bardeen-Cooper-Schrieffer theory predicts Δ = *Ak*_B_*T*_CDW_ with *A* = 1.764 in the weak coupling limit and, to our knowledge, typically no higher than 3 in the strong-coupling regime and *k*_B_ as the Boltzmann constant. ARPES results commonly estimate a 100-meV separation between conduction and valence bands in the CDW phase, justified by a semiconducting gap of 75 to 150 meV (*52*, *59*, *67*). Given these considerations and our semimetallic model, we thus use Δ(0) = 40 meV as a reasonable estimate of the CDW order parameter (observed separation in the CDW minus the preexisting semiconducting gap), which we find to reproduce the Lifshitz transition at 2 GPa, and to be in accordance with TiSe_2_ being in the strong-coupling regime (*A* ≈ 2.3). Under pressure, we scale Δ(*P*) = Δ(0) × *T*_CDW_(*P*)/*T*_CDW_(0) (*28*). Using these steps, we obtain a model with minimal degrees of freedom that can account for the CDW. The electronic structure of Cu*_x_*TiSe_2_ is calculated by shifting the chemical potential in accordance with the electron doping from the Cu intercalation (*58*, *59*) and reducing Δ proportional to *T*_CDW_ (*3*) (see section S6 for details).

In summary, after calibrating the DFT to quantum oscillation measurements above *P*_CDW_, we consider only two parameters in our tight-binding model: the magnitude of the CDW gap Δ(0) and the linear pressure shift of the bands. These two parameters are determined by two fixed points: the size of the Fermi surface at ambient pressure and the pressure at which the Lifshitz transition is observed. All other quantitative agreement follows naturally, including *F*_γ_(*p*), the presence of *F*_β_ and its pressure dependence, and the persistence of *F_α_*. These are independent confirmations for our model. The CDW bandstructure shown in Fig. 4 may be topological as the bands are inverted.

## Supplementary Materials

### This PDF file includes:

Sections S1 to S6 Figs. S1 to S11 References
