# Supplementary material for: Lifshitz transition enabling superconducting dome around a charge-order critical point
Source: Sci Adv. 2024 Jul 5;10(27):eadl3921. doi: 10.1126/sciadv.adl3921 (PMC11225790; doi:10.1126/sciadv.adl3921)
Supplement: Supplementary file 1 — Sections S1 to S6 Figs. S1 to S11 References [file sciadv.adl3921_sm.pdf]

Science Advances
AAAS

Supplementary Materials for

Lifshitz transition enabling superconducting dome around a charge-order critical point

Roemer D. H. Hinlopen *et al.*

Corresponding author: Sven Friedemann, sven.friedemann@bristol.ac.uk

Sci. Adv. **10**, eadl3921 (2024)
DOI: 10.1126/sciadv.adl3921

This PDF file includes:

Sections S1 to S6
Figs. S1 to S11
References

SI. SAMPLE PREPARATION AND CHARACTERIZATION

Single crystals of $1T$-$TiSe_2$ were grown by chemical vapor transport (CVT) using iodine as a flux. High purity titanium (99.98 %), selenium (99.99 %) and iodine (99.998 %, anhydrous) were used as the starting materials. To achieve a low doping/defect density, a slight selenium excess was included (18) and the growth temperature was set to 570 °C (68). Native defects in $TiSe_2$ include selenium vacancies or substitution with iodine, and titanium intercalation (69), resulting in electron doping which systematically reduces the charge density wave transition temperature (18). Based on previous characterization of our samples, we find the expected charge density wave transition of 202 K, and estimate an electron doping of no more than 0.1 % (30).

Studies of quantum oscillations and superconductivity were conducted on the very same sample #1 in a moissanite anvil pressure cell. The quantum oscillation study was conducted upon increasing pressure. The resistance measurements observing superconductivity were completed afterward upon decompression of the cell.

No hysteresis of $T_{\mathrm{CDW}}(P)$ is present as can be seen from Figure S1 and Figure 1 of the main text. At low pressures, we identify T_{CDW} with a large minimum in the derivative $\mathrm{d}R/\mathrm{d}T$ (28). Naturally, this signature becomes weaker close to the critical pressure of the CDW. At 3.3 GPa, we only identify a weak minimum similar to previous studies at this pressure (28) where such a minimum was corroborated with stronger signatures in magnetoresistance.

Additional evidence for superconductivity in $TiSe_2$ stems from the suppression of the resistance drop in magnetic fields. In Figure S2, we show representative magnetoresistance measurements at 2.1 GPa, immediately after the Lifshitz transition enabling the superconductivity. We used field sweeps to map the superconducting phase and find the characteristic suppression of the zero-resistance state at magnetic fields above 0.2 T. The presence of superconductivity starting at 2 GPa is in agreement with previous observation (25). Compared to this previous study, we observe a higher T_c and higher H_{c2} value (yet remain well below the Pauli limit), which we assign to the improved hydrostaticity and sample homogeneity in our experiment. Combined, we believe these observations constitute comprehensive evidence for superconductivity in $TiSe_2$.

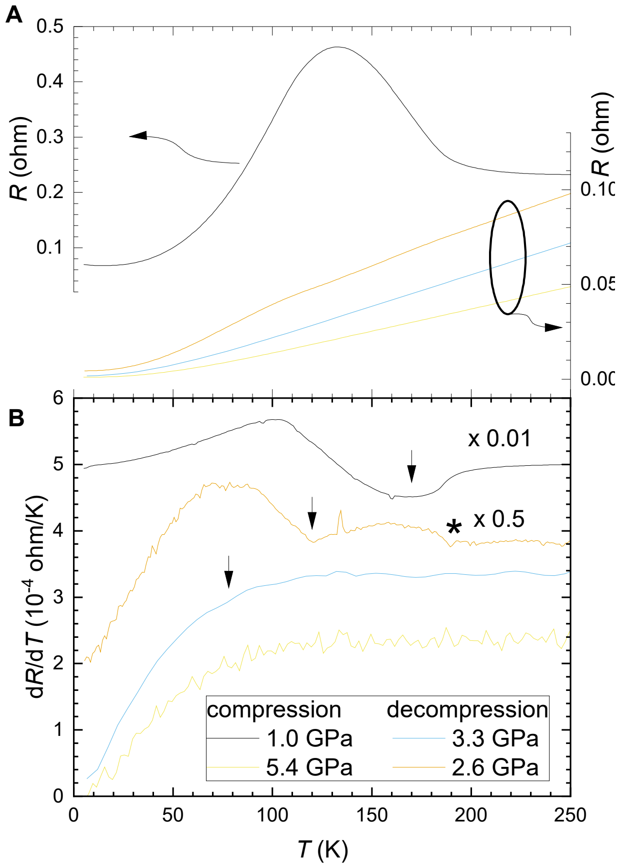

FIG. S1. **CDW signature in resistivity of sample #1.** Zero-field resistance (a) and derivative (b). The CDW transition temperature was extracted as the minimum in the derivative of the resistivity, indicated by black arrows and is included in Fig. 1(b) of main text. Data at 1.1 GPa and 2.6 GPa are offset and scaled in (b) for visibility. The asterisk indicates the temperature which we associate with the freezing of the pressure medium (pentane:isopentane (70)) similar to earlier work (28).

SII. QUANTUM OSCILLATIONS AT HIGH PRESSURE

To analyse the quantum oscillations, a background contribution needs to be removed from the raw magnetoresistance data. The background arises mostly from magnetoresistance but may include Hall effect contributions due to the alignment of sample contacts. Both magnetoresistance and Hall effect vary significantly with pressure with the band structure of $TiSe_2$ changing from a single band to multiple bands with significantly increased carrier concentrations. The background subtraction aims to centre the oscillations at zero resistance

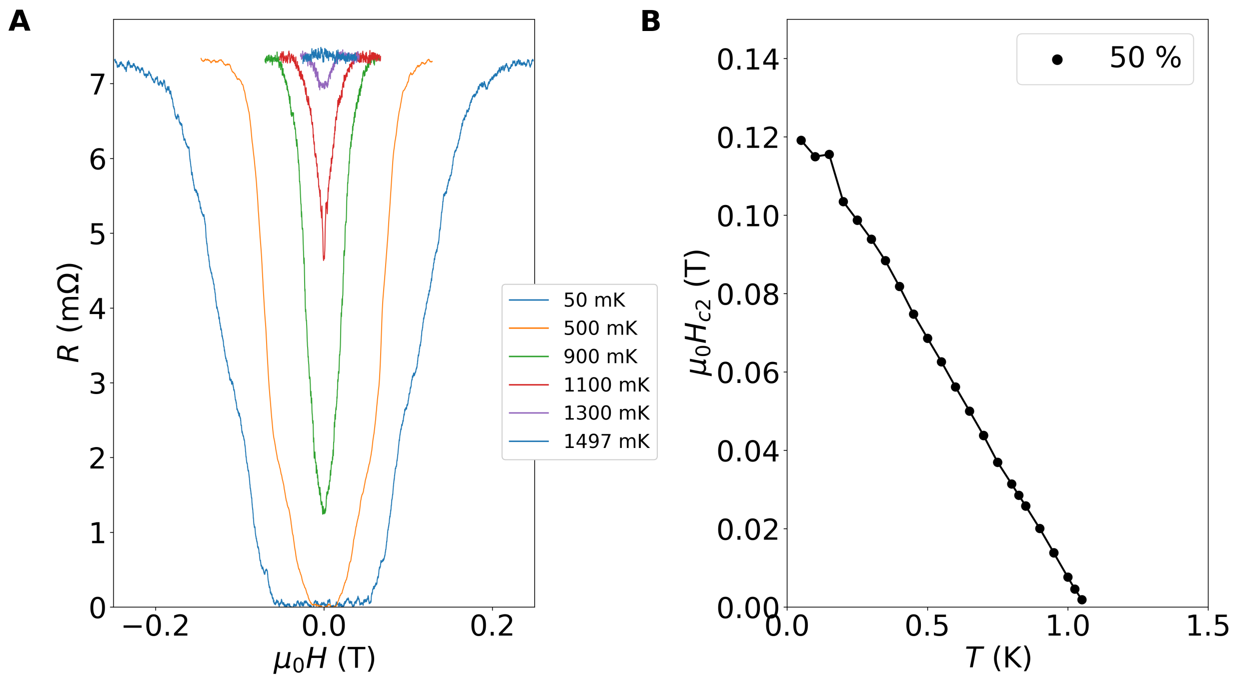

FIG. S2. **Suppression of superconductivity in magnetic fields.** (a) Resistance measurements as a function of magnetic field at selected temperatures at 2.1 GPa using 5 μA. (b) The upper critical field $H_{c2}(T)$ is extracted from the midpoint (50 %) of all resistive transitions measured at 2.1 GPa.

whilst avoiding the introduction of spurious oscillations; both would appear as artefacts in the subsequent Fourier transform analysis. Figure S3 shows the raw resistance data obtained beside the associated background subtractions at several pressures. We see that linear subtractions do not centre the oscillations about zero, but parabolic and cubic fits suffice. The parabolic and cubic subtractions yield very similar results. An artificial frequency can arise that is associated with the magnetic field range between the extrema of a cubic function. At worst, and as shown by the data at 2.0 GPa, one of the turning points appears within the region of interest. In this instance, we use the maximum magnetic field applied (B_{max}=35 T) and the difference between the turning points ($\Delta B = 38.5 - 29.2 = 9.3$ T) to estimate the lowest reliable frequency ($F_{min} = B^2_{max}/\Delta B$). In this worst-case scenario, F_{min} equals 132 T. Thus, frequencies above $\approx$ 150 T are reliably extracted by our method. The lowest frequency oscillations associated with F_α are visible in the raw data.

At pressures above 4.5 GPa, we observe a low frequency (F_L) peak between 120 − 175 T (shown in Figure S4(a)). This frequency is only slightly above F_{min}. Figure S4(b) shows

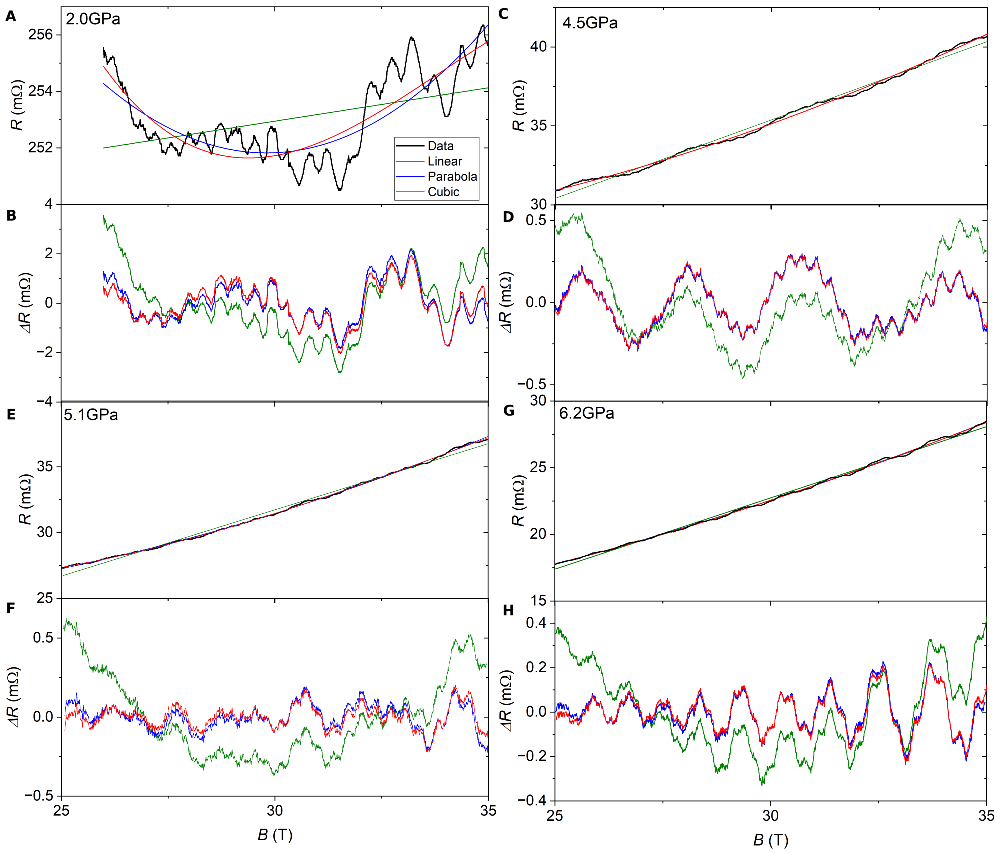

FIG. S3. **Background subtraction in quantum oscillation measurements.** Raw magnetoresistance data both before and after subtraction of fitted polynomial backgrounds at various pressures and at lowest temperatures.

that for 5.1 GPa and 6.2 GPa, the temperature dependence of the amplitude of F_{L} does not follow Lifshitz-Kosevich behaviour, whilst for 4.5 GPa and 5.5 GPa, we find Lifshitz-Kosevich behaviour with masses of $0.60(2)\,m_e$ and $0.54(3)\,m_e$. Though the Lifshitz-Kosevich behaviour at 4.5 GPa and 5.5 GPa is convincing, the absence of Lifshitz-Kosevich behaviour at intermediate pressures combined with the proximity of F_L to F_{min} highlights uncertainties around F_{L}. We omit F_{L} from Figure 3 of the main text due to the inconclusive behaviour and the small size of the orbit and thus minor contribution to the electronic states. Quantum oscillation measurements over an extended field range would be required to ascertain the nature of F_{L}, which, if confirmed, may correspond to either a hole-like neck orbit at the top of the Brillouin zone at A, or an electron-like neck orbit in the $k_z = 0$ plane at M.

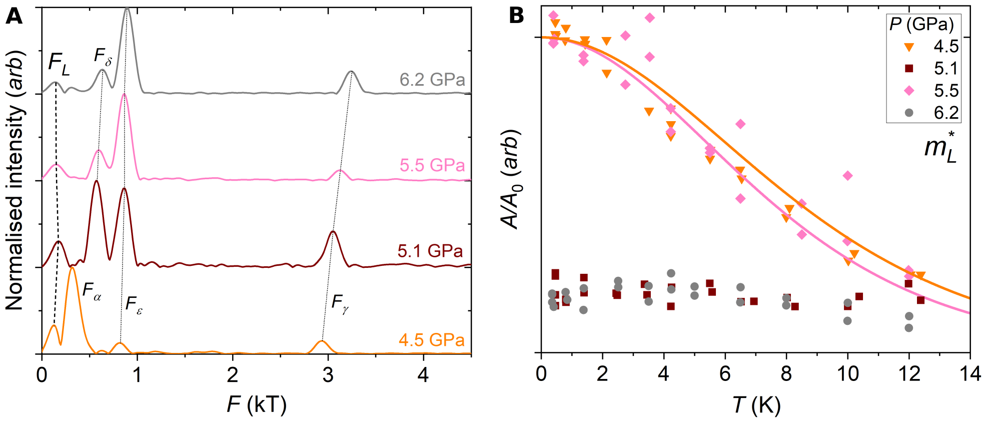

FIG. S4. **Lowest frequency oscillation.** (a) FFTs at high pressure highlighting the presence of the lowest frequency oscillation observed in measurements (F_L). (b) Lifshitz-Kosevich curves from which the effective mass of F_L (m^*_L) can be determined. At 5.1 GPa and 6.2 GPa, we only observe noise and cannot determine an associated mass. These two curves are normalised to the same A_0 as used at 4.5 GPa.

SIII. POSSIBLE MAGNETIC BREAKDOWN ORBITS

Our DFT calculations quantitatively capture the evolution of quantum oscillations under hydrostatic pressure in $TiSe_2$. Nevertheless, there remain a number of unexplained frequencies in the pressure range 3.7-5.0 GPa in Figure 3 of the main text. We here discuss their possible origin through magnetic breakdown inside the CDW, particularly when the gap becomes smaller at high pressures (55).

The experimentally detected α, γ and δ branches show a smooth evolution of the frequency and effective mass under pressure and are well identified based on the DFT results. Yet, the ϵ branch is not expected in our model below P_{CDW}. In fact, we trace the ϵ orbit into the CDW regime down to 4 GPa. This may be the result of magnetic breakdown through the small CDW gap which can produce orbits of the underlying non-reconstructed Fermi surface. Indeed, our model indicates multiple possibilities exist for magnetic breakdown between the β and α orbits in $TiSe_2$ near the end of the CDW phase. This includes an orbit that traces the ϵ pocket of the unreconstructed Fermi surface, which may explain the observation of F_ϵ inside the CDW phase in Figure 3 of the main text.

At 3.7 GPa, we observe a fifth frequency (open triangle in Figure 3 of the main text). At this pressure, we assign 1.0 kT to the β branch and 1.7 kT to magnetic breakdown. In

this scenario, 1.7 kT is the sum of F_β and F_ϵ, which both originate from electron-like orbits in the DFT calculations indicating a possibility for magnetic breakdown to result in a sum frequency. Furthermore, we observe a high mass $m_{1.7\mathrm{kT}} = 2.0(3)$ $\mathrm{m_0}$, which is consistent with the sum of masses $m_{1.7\mathrm{kT}} = m_\beta + m_\epsilon$.

SIV. DENSITY FUNCTIONAL THEORY UNDER HYDROSTATIC PRESSURE

In order to evolve the electronic structure of $TiSe_2$ under pressure, we require the pressure-dependent lattice parameters. We start by optimising the internal degrees of freedom at constant unit cell volume V_{uc}. We use Wien2k (54) excluding spin-orbit coupling with 3000 k-points, $Rk_{max} = 10$ and an 11 % muffin tin radius reduction. The lattice is fully defined by $(V_{uc}, c/a, z)$, where c/a is the ratio of the in-plane and out-of-plane lattice parameter and z is the fraction of c by which the Se atoms are alternating above and below the plane. We minimise the total energy as a function of c/a and z for fixed V_{uc}.

The results of these calculations are optimised lattice parameters as a function of V_{uc}. In order to convert V_{uc} to pressure we fit a Birch-Murnaghan equation of state:

$$E = E_0 + \left((x-1)^3 \frac{dB}{dP}_0 + (x-1)^2 (6-4x) \right) \frac{9}{16} V_0 B_0 \quad \text{with} \quad x = \left(\frac{V_0}{V_{uc}} \right)^{2/3} \tag{S1}$$

Here, V_0 is a reference volume where $E = E_0$, B_0 is the bulk modulus and P pressure. $E_0, V_0, B_0, dB/dP_0$ are fit parameters given in Figure S5. To convert volume to pressure, we use:

$$P = \frac{3}{2} B_0 \left(\frac{V_0}{V_{uc}}^{7/3} - \frac{V_0}{V_{uc}}^{5/3} \right) \left(1 + \frac{3}{4} \left(\frac{dB}{dP}_0 - 4 \right) \left(\frac{V_0}{V_{uc}}^{2/3} - 1 \right) \right) \tag{S2}$$

We thus obtain the lattice parameter evolution as a function of pressure. These results are compared in figure Figure S6 to X-ray diffraction measurements under pressure.

At each pressure, we perform a detailed calculation using 10,000 k-points and including spin-orbit coupling. Going forward, we use these detailed calculations at the optimised c/a and z obtained via this procedure.

Next, we shift the bands to match the quantum oscillation frequencies observed above $P_{\mathrm{CDW}} = 5$ GPa. These shifts are -150 meV for the small hole band, -80 meV for the large hole band and +55 meV for the electron bands. The direction of the shift in each case reduces the

semimetallic overlap compared to the DFT prediction and preserves charge neutrality. We keep these bandshifts fixed at 5 GPa and add a pressure dependent shift to the hole bands of +12 meV/GPa and to the electron bands of -12 meV/GPa to fit the ambient pressure quantum oscillation frequency (see Figure S11).

The resulting band structures are fit with a tight binding model. We use nearest neighbour hopping parameters (t_0 and t_1) for both hole bands and 3 nearest neighbours (t_0, t_1, t_2, t_3) for the electron bands following the notation in Ref. (65). The resulting hole pockets are circular, whereas the electron pockets are elliptical. The parameters are available in Ref. (**?**). We perform our tight binding fits only in the $k_z = 0$ plane for the holes and $k_z = \pm\pi/c$ plane for the electrons because the CDW $\vec{Q}$ connects pockets in these planes. In particular, we fit to a detailed evaluation of $\epsilon(k)$ along high-symmetry lines: ΓK and ΓM for the hole pockets, and LA and LH for the electron bands up to ± 150 meV around the Fermi level, see Figure S7.

Our tight-binding fits result in an overestimate of the band overlap at ambient pressure in Figure 4 of the main text below ~ 1 GPa. Our tight binding model captures the p_z orbital contribution to the hole bands well (Figure S7(f)) at high pressures, but below $\sim$1 GPa it is influenced by the p_{xy} contributions from the DFT calculations (Figure S7(d)), which we argue are absent in experiment (cf. Fig. 3 of main text). Using the data shown in Figure S7, we estimate a band overlap of $\sim$ 160 meV at ambient pressure which increases to $\sim$ 730 meV at 6 GPa. The model obtains this increase in semimetallic overlap with hydrostatic pressure primarily through an increased bandwidth of the p_z anti-bonding band as the c-axis shrinks. The ambient pressure band overlap is consistent with the ambient pressure Hall measurements extrapolated from above T_{CDW}, which predict a band overlap of 145 meV at $T = 0$ (30).

Overall, the model has only 5 degrees of freedom: pressure-independent band energy shifts at 5 GPa for each of the 3 bands. 1 band-independent linear-in-pressure energy shift. These values remain well below the band energies. Finally, we use $\Delta(0) = 40$ meV.

SV. TIGHT BINDING FITS AND THE SHAPE OF THE ELECTRON ORBIT

The shape of the electron orbit at L is not perfectly captured by 3 nearest neighbour hopping parameters. The resulting tight binding fit has a more rounded elliptical shape than

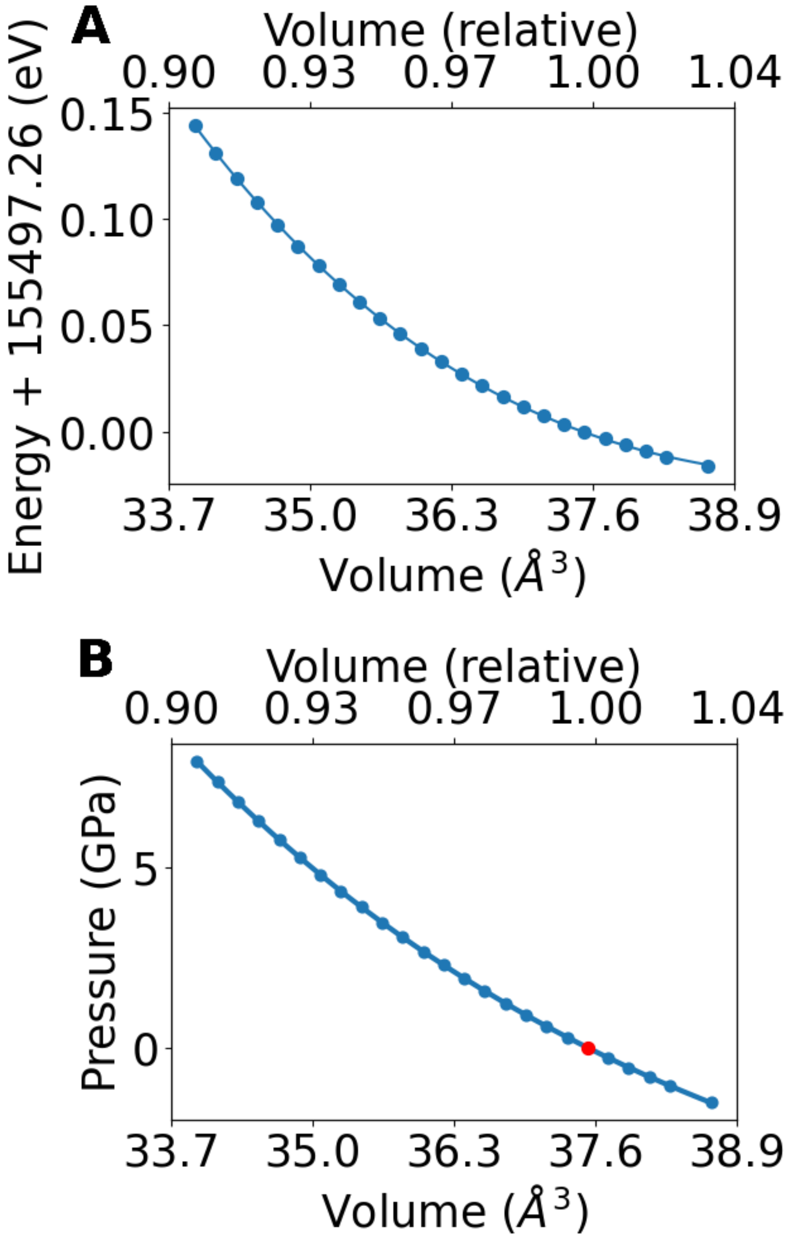

FIG. S5. **Birch-Murnaghan fits.** (a) Total energy from DFT calculations with varying unit cell volume. For each point, the internal degrees of freedom are optimized. 0 energy is assigned to the experimental ambient pressure volume. The line represents the Birch-Murnaghan equation of state fit (Eq. (S1)). (b) Using the Birch-Murnaghan parameters, we derive the pressure dependence of the unit cell volume using Eq. (S2). The fit parameters are $V_0 = 75.7503\,\text{Å}^3$, $E_0 = -155\,496.877\,79\,\text{eV}$, $B_0 = -0.0504\,\text{eV}/\text{Å}^3$, $dB/dP = 1.48$. The volume at ambient pressure is $37.535\,\text{Å}^3$ and marked in red.

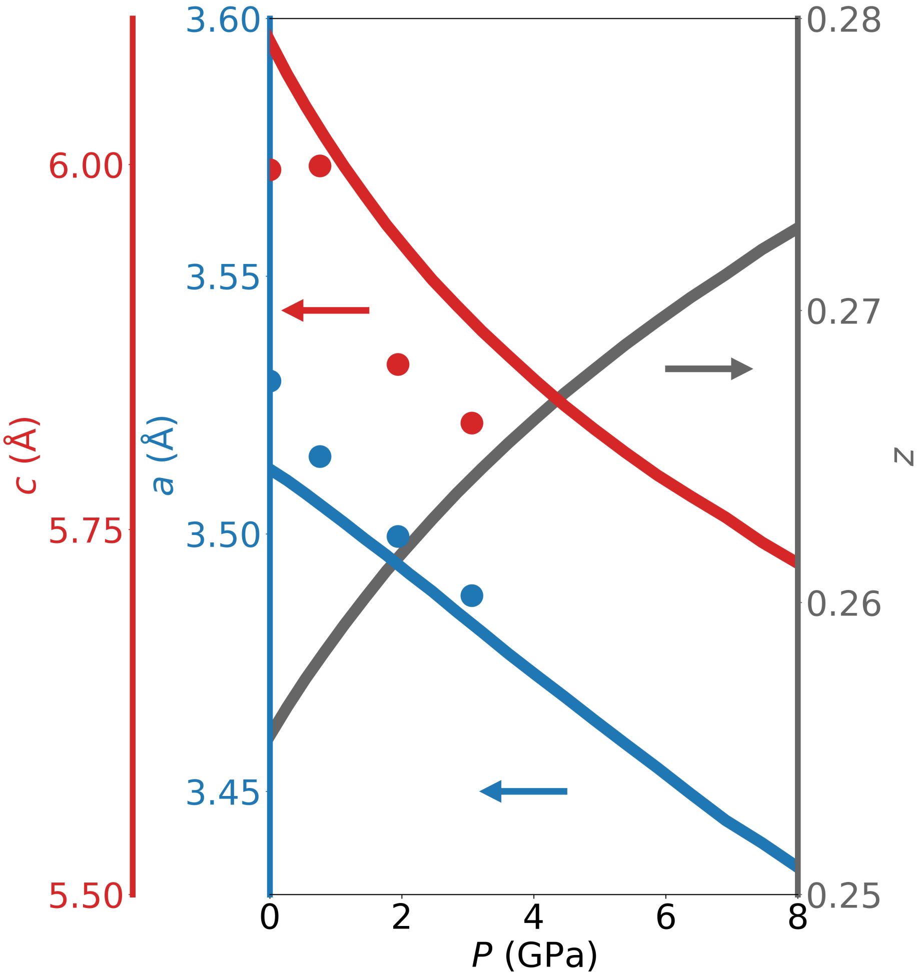

FIG. S6. **Lattice parameter change with pressure.** Lattice parameters a and c as a function of pressure obtained by energy minimization within DFT. Each Se atom is $\pm cz$ out of the plane. Lines indicate theoretical, points experimental results. Ambient pressure X-ray diffraction results from Ref. (60), pressure dependence from Ref. (26).

the sharp DFT pocket. For the main text, we fit the energy dispersion along high symmetry lines and therefore fit the axes of the elliptical pocket. This allowed us to identify the hybridisation of electron and hole pockets accurately. Even though the bands were shifted to match the experimental quantum oscillation frequencies above 5 GPa, the round shape of the tight binding fit compared to the more pointy DFT band results in an overestimate of F_ϵ by $\sim$200 T in our tight binding model (see Figure S9(b)). This offset directly translates into an overestimate of F_β by up to 600 T as F_β is a combined orbit including sections of all three electron pockets.

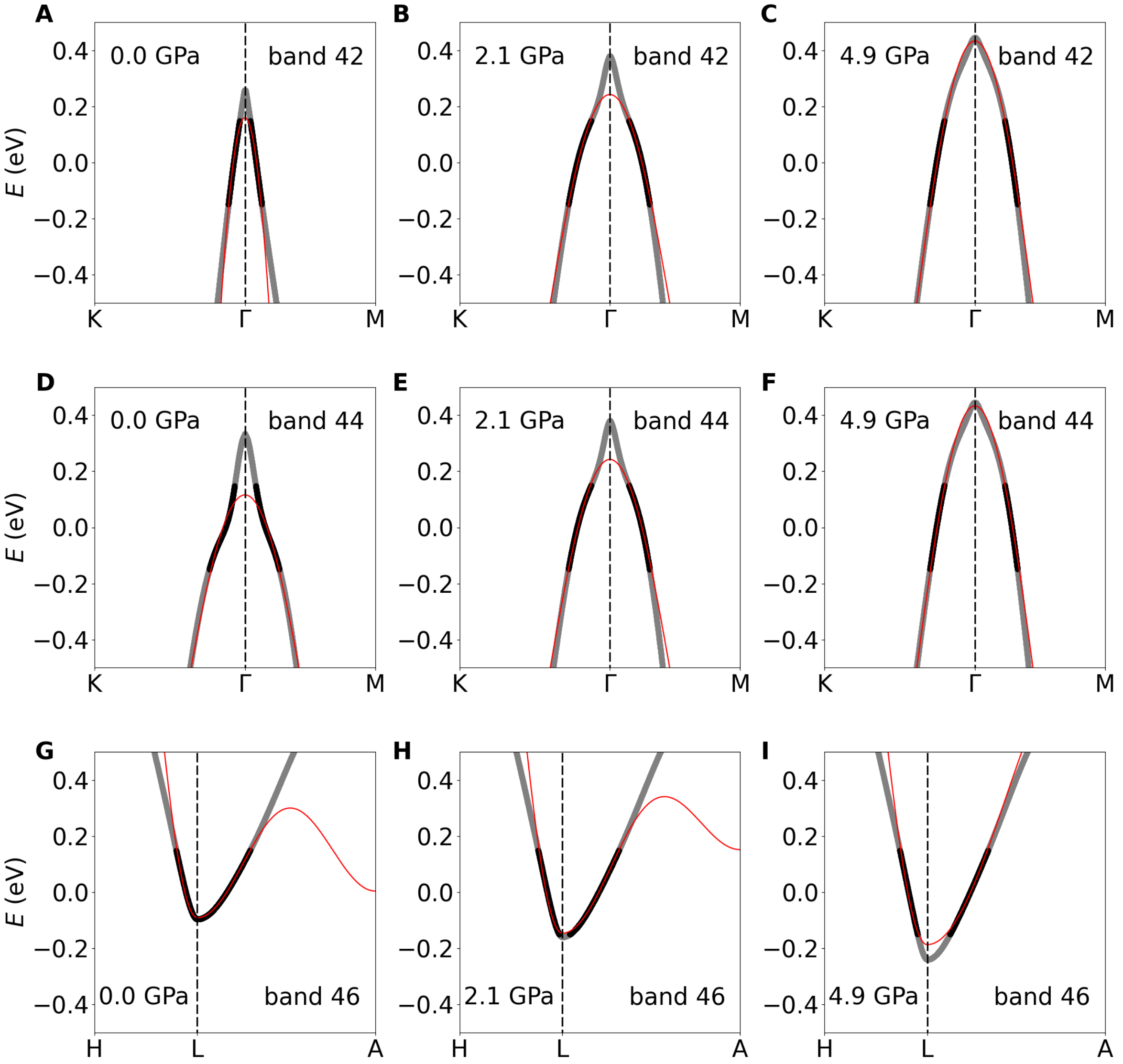

FIG. S7. **Selected tight binding fits**. (a)-(c) Pressure dependence of the band 42 (small hole band) tight binding fit. Grey represents DFT energies after the band shifts described in the text, black the DFT energy range of fits, and red lines the resulting tight binding fit. (d)-(f) Same for band 44 (the large hole band). (g)-(i) Same for band 46 (electron band).

In order to prove this indeed explains the offset of F_β and F_ϵ, we change the parametrisation of the tight binding model. We shorten the long axis of the electron bands in the tight binding model in order to match the DFT quantum oscillation frequencies rather than axes above P_{CDW}. As shown in Figure S9(a), the result matches F_ϵ by construction. We subse-

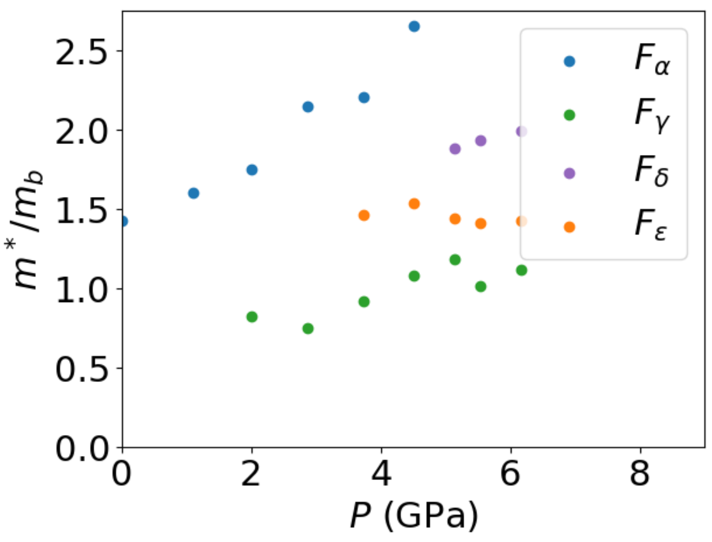

FIG. S8. **Band mass enhancement factors**. This figure shows the experimental quantum oscillation mass m^* compared to the DFT derived band mass m_b. In particular, the ratio increases towards P_{CDW} for F_α whereas for F_γ the ratio is close to 1.

quently implement the CDW model as before and observe F_β reduces to the experimental value whereas all other frequencies inside the CDW regime remain unaffected. The caveat is that the k-space separation between the electron and hole pockets in the CDW regime is enlarged. Consequently, although we have corrected the predicted quantum oscillation frequencies, we have introduced an error in the location of the predicted Lifshitz transition. Therefore, we show the original model which overestimates F_β and F_ϵ in the main text. This construction shows that the mismatch between the experimental and calculated F_β and F_ϵ is an artifact of the tight-binding fit procedure.

SVI. DOPING DEPENDENCE AND DEGREES OF FREEDOM FOR THE DFT MODEL

First, we check the evolution of lattice parameters from Cu_xTiSe_2 X-ray diffraction results in Ref. (60). These indicate the lattice parameter change induced by 5 % Cu intercalation is 0.2 % in c-direction and undetectable in-plane, which is much less than the lattice parameter changes shown in Figure S6 for the pressure dependence. We therefore neglect changes in the lattice parameters under Cu intercalation.

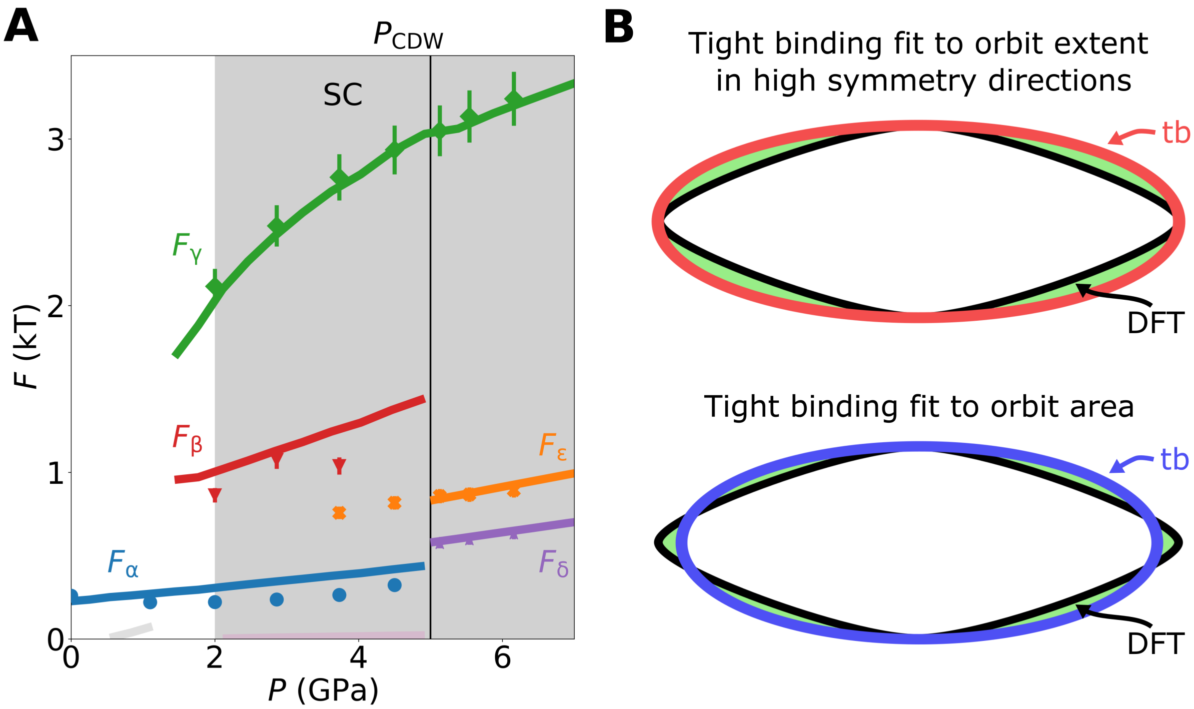

FIG. S9. **Influence of the electron pocket shape**. In Figure 3 of the main text we use tight binding calculations fit to the semi-axes of the L pocket at the cost of increased quantum oscillation frequencies F_β and F_ϵ. We here show the same calculation and results, except the tight binding model is optimised to fit the area of the L pocket and hence matches F_ϵ as predicted by DFT. Consequently, F_β and F_ϵ shift as well as the position of the Lifshitz transition at 2 GPa. (a) Quantum oscillation frequencies. (b) Schematic representation of the two models used. The red line shows a rounded tight binding fit to the axes of a pointy DFT pocket (black line) which overestimates the area as in the main text, whereas the shortened axis used in panel (a) (blue line, bottom) preserves the cross sectional area of the pointy ellipse.

In order to calculate the electronic structure of Cu_xTiSe_2 we require two additional ingredients. The first ingredient is the charge transfer from the Cu to the $TiSe_2$ planes. We use 0.45 electrons/Cu as determined by supercell DFT calculations (56).

The second ingredient is a reconstruction of the out-of-plane shape of the Fermi surface to obtain the total carrier density to compare to the Cu intercalation. The absence of the hole-like quasi-2D neck pockets is well established at ambient pressure from ARPES (e.g. (52)) and confirmed in the bulk by angle-dependent quantum oscillation experiments, magnetotransport and specific heat (30). Hence, we assume spherical Fermi surfaces as

established by quantum oscillations at ambient pressure. At ambient pressure the Fermi surface is known in detail from Ref. (30) and shows the actual Fermi surface volume is only 17 % of a full 2D cylinder. Using our DFT results for the electron band, we estimate this fraction increases by 15 percentage points at $\mu = 100\,\text{meV}$. In order to maintain minimal degrees of freedom, we therefore obtain the filling fraction n of the bands via:

$$n = \frac{A}{A_{BZ}}(0.17 + 0.0015\mu) \tag{S3}$$

Here, A is the cross sectional area of the pocket at constant k_z, A_{BZ} is the cross section of the Brillouin zone at ambient pressure. μ is the chemical potential in meV relative to the undoped case derived from the pressure dependent model discussed above. We take $\Delta(x = 0) = 40\,\text{meV}$ in line with our pressure results and take $\Delta(x)$ to be proportional to $T_{\text{CDW}}(x)$ as shown in Figure 5 of the main text. The CDW gap is fully determined by x and independent of μ, k or orbital.

Using the conversion between the Fermi surface area and Cu content we obtain a chemical potential evolution as a function of x. This result is shown in figure Figure S10. Hall effect measurements analysed with $n_H = 1/R_H q$ are consistent with the resulting model (57). We also find a shift of 60 meV in chemical potential at $x = 4$ % as shown inFigure S10 matches previous ARPES results (58, 59).

As a result of the additional degrees of freedom as well as lack of quantum oscillation data under Cu intercalation, a larger uncertainty exists in the electronic structure evolution under doping than pressure. No matter the precise choice for these degrees of freedom, however, the hole pocket emerges at lower x than the CDW QCP as a result of the k-space separation of the electron and hole bands. We have attempted to choose natural values for each of the degrees of freedom throughout rather than fitting the result. The location of the Lifshitz transition at $x = 3$ % is therefore in good agreement with the observed start of the superconducting dome under Cu intercalation.

In Figure S11 we demonstrate the insensitivity of the ambient pressure result to the precise value of $\Delta(0)$ and small doping variation. Only minor variations of F_α and n are found across μ shifts by $10\,\text{meV}$ across the range of $\Delta(0)$ explored. This highlights that our results are not highly sensitive to the precise value of $\Delta(0)$ as only a minor effect on the ambient pressure quantum oscillation frequency is observed which could be accounted for by small adjustments of the chemical potential – similar to the pressure results. Rather, our

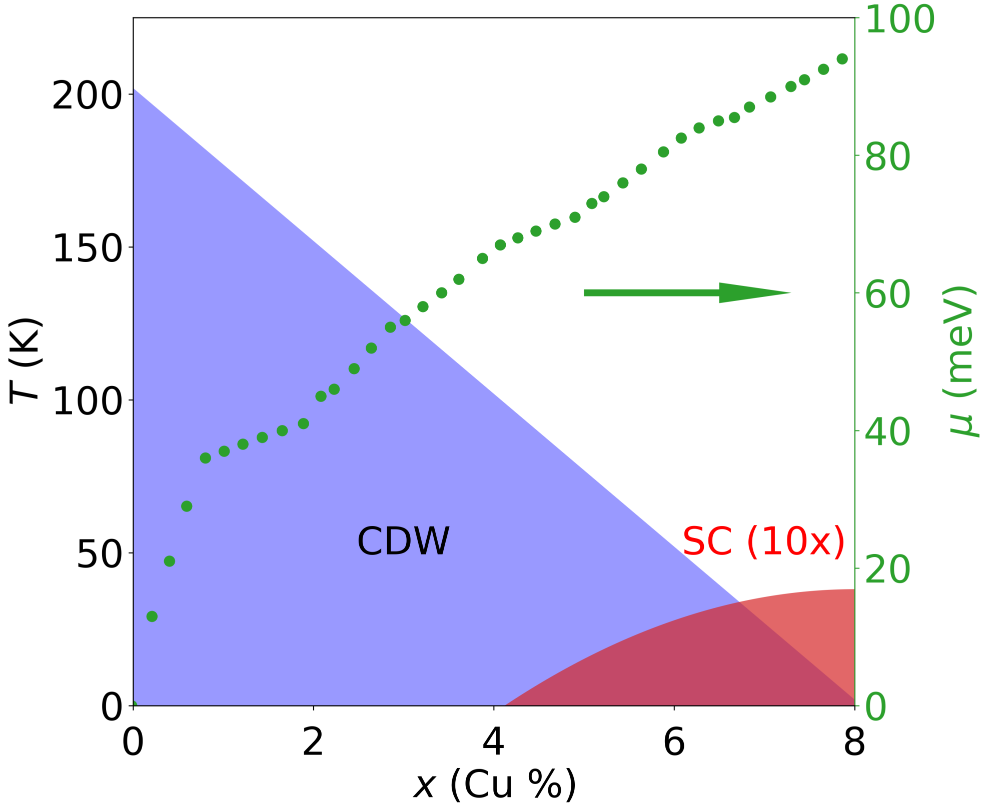

FIG. S10. **Chemical potential with Cu intercalation**. The shaded areas indicate the extent of the CDW and superconducting order. See text for details on the calculation.

model robustly reproduces the experimental quantum oscillation frequencies.

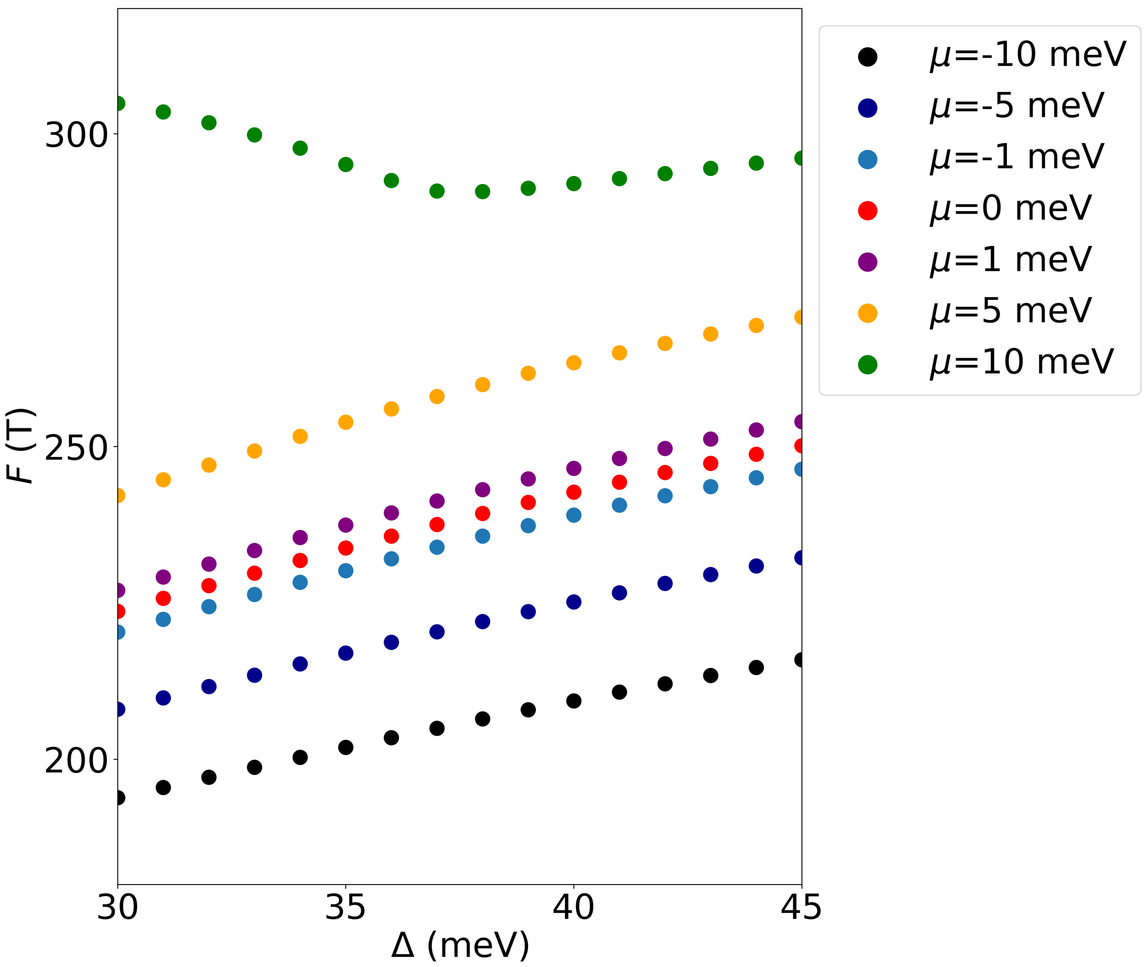

FIG. S11. **Sensitivity of ambient pressure, zero doping result.** The figure shows the change in quantum oscillation frequency at ambient pressure with $H \parallel c$ upon variation of the gap size and chemical potential (doping). Shifting the chemical potential results in a 3.7 T/meV shift in quantum oscillation frequencies, or doping of -5.1e24 1/meV/m^3, which is negative because an increase in chemical potential electron dopes the material. Changing the gap size has a relatively modest influence of 1.6 T/meV. Evidently, the chemical potential shifts predominantly defines the ambient pressure quantum oscillation and pocket size.

REFERENCES AND NOTES

1. N. D. Mathur, F. M. Grosche, S. R. Julian, I. R. Walker, D. M. Freye, R. K. W. Haselwimmer, G. G. Lonzarich, Magnetically mediated superconductivity in heavy fermion compounds. *Nature* **394**, 39–43 (1998).

2. T. Shibauchi, A. Carrington, Y. Matsuda, A quantum critical point lying beneath the superconducting dome in iron-pnictides. *Annu. Rev. Condens. Matter Phys.* **5**, 113–135 (2014).

3. E. Morosan, H. W. Zandbergen, B. S. Dennis, J. W. G. Bos, Y. Onose, T. Klimczuk, A. P. Ramirez, N. P. Ong, J. V. Cava, Superconductivity in Cu_xTiSe_2, *Nat. Phys.* **2**, 544–550 (2006).

4. G. Gruner, *Density Waves in Solids* (Addison-Wesley Pubishing Company, 1994).

5. Q. Si, R. Yu, E. Abrahams, High-temperature superconductivity in iron pnictides and chalcogenides. *Nat. Rev. Mater.* **1**, 16017 (2016).

6. P. Monthoux, D. Pines, G. G. Lonzarich, Superconductivity without phonons. *Nature* **450**, 1177–1183 (2007).

7. L. Taillefer, Scattering and pairing in cuprate superconductors. *Annu. Rev. Condens. Matter Phys.* **1**, 51–70 (2010).

8. T. Vuletić, P. Auban-Senzier, C. Pasquier, S. Tomić, D. Jérome, M. Héritier, K. Bechgaard, Coexistence of superconductivity and spin density wave orderings in the organic superconductor $(TMTSF)_2PF_6$. *Eur. Phys. J. B.* **25**, 319–331 (2002).

9. M.-A. Measson, Y. Gallais, M. Cazayous, B. Clair, P. Rodiere, L. Cario, A. Sacuto, Amplitude Higgs mode in the $2H$−$NbSe_2$ superconductor. *Phys. Rev. B* **89**, 060503 (2014).

10. X. Xi, L. Zhao, Z. Wang, H. Berger, L. Forro, J. Shan, K. F. Mak, Strongly enhanced charge-density-wave order in monolayer $NbSe_2$. *Nat. Nanotechnol.* **10**, 765–769 (2015)

11. O. Moulding, I. Osmond, F. Flicker, T. Muramatsu, S. Friedemann, Absence of superconducting dome at the charge-density-wave quantum phase transition in $2H$−$NbSe_2$. *Phys. Rev. Res.* **2**, 043392 (2020).

12. C. Putzke, J. Ayres, J. Buhot, S. Licciardello, N. E. Hussey, S. Friedemann, A. Carrington, Charge order and superconductivity in underdoped $YBa_2Cu_3O_{7-\delta}$ under Pressure. *Phys. Rev. Lett.* **120**, 117002 (2018).

13. C. C. Tam, M. Zhu, J. Ayres, K. Kummer, F. Yakhou-Harris, J. R. Cooper, A. Carrington, and S. M. Hayden, Charge density waves and Fermi surface reconstruction in the clean overdoped cuprate superconductor $Tl_2Ba_2CuO_{6+\delta}$. *Nat. Commun.* **13**, 570 (2022).

14. C. C. Tam, J. Choi, X. Ding, S. Agrestini, A. Nag, M. Wu, B. Huang, H. Luo, P. Gao, M. García-Fernández, L. Qiao, K.-J. Zhou, Charge density waves in infinite-layer $NdNiO_2$ nickelates. *Nat. Mater.* **21**, 1116–1120 (2022).

15. F. H. Yu, D. H. Ma, W. Z. Zhuo, S. Q. Liu, X. K. Wen, B. Lei, J. J. Ying, X. H. Chen, Unusual competition of superconductivity and charge-density-wave state in a compressed topological kagome metal. *Nat. Commun.* **12**, 3645 (2021).

16. F. Du, S. Luo, B. R. Ortiz, Y. Chen, W. Duan, D. Zhang, X. Lu, S. D. Wilson, Y. Song, H. Yuan, Pressure-induced double superconducting domes and charge instability in the kagome metal KV_3Sb_5. *Phys. Rev. B* **103**, L220504 (2021).

17. W. Zhang, X. Liu, L. Wang, C. W. Tsang, Z. Wang, S. T. Lam, W. Wang, J. Xie, X. Zhou, Y. Zhao, S. Wang, J. Tallon, K. T. Lai, S. K. Goh, Nodeless superconductivity in kagome metal CsV_3Sb_5 with and without time reversal symmetry breaking. *Nano Lett.* **23**, 872–879 (2023).

18. F. Di Salvo, D. Moncton, J. Waszczak, Electronic properties and superlattice formation in the semimetal $TiSe_2$. *Phys. Rev. B* **14**, 4321 (1976).

19. D. Jerome, T. M. Rice, W. Kohn, Excitonic insulator. *Phys. Rev.* **158**, 462–475 (1967).

20. K. Rossnagel, On the origin of charge-density waves in select layered transition-metal dichalcogenides. *J. Phys. Condens. Matter* **23**, 213001 (2011).

21. S. Hellmann, T. Rohwer, M. Kalläne, K. Hanff, C. Sohrt, A. Stange, A. Carr, M. M. Murnane, H. C. Kapteyn, L. Kipp, M. Bauer, K. Rossnagel, Time-domain classification of charge-density-wave insulators. *Nat. Commun.* **3**, 1069 (2012).

22. A. Kogar, M. S. Rak, S. Vig, A. A. Husain, F. Flicker, Y. I. Joe, L. Venema, G. J. MacDougall, T. C. Chiang, E. Fradkin, J. van Wezel, P. Abbamonte, Signatures of exciton condensation in a transition metal dichalcogenide. *Science* **358**, 1314–1317 (2017).

23. H. Hedayat, C. J. Sayers, D. Bugini, C. Dallera, D. Wolverson, T. Batten, S. Karbassi, S. Friedemann, G. Cerullo, J. van Wezel, S. R. Clark, E. Carpene, E. Da Como, Excitonic and lattice contributions to the charge density wave in $1T-TiSe_2$ revealed by a phonon bottleneck. *Phys. Rev. Res.* **1**, 023029 (2019).

24. J. Ishioka, Y. H. Liu, K. Shimatake, T. Kurosawa, K. Ichimura, Y. Toda, M. Oda, S. Tanda, Chiral charge-density waves. *Phys. Rev. Lett.* **105**, 176401 (2010).

25. A. F. Kusmartseva, B. Sipos, H. Berger, L. Forro, E. Tutis, Pressure induced superconductivity in pristine $1T-TiSe_2$. *Phys. Rev. Lett.* **103**, 236401 (2009).

26. Y. I. Joe, X. M. Chen, P. Ghaemi, K. D. Finkelstein, G. A. de la Pena, Y. Gan, J. C. T. Lee, S. Yuan, J. Geck, G. J. MacDougall, T. C. Chiang, S. L. Cooper, E. Fradkin, P. Abbamonte, Emergence of charge density wave domain walls above the superconducting dome in $1T$-$TiSe_2$. *Nat. Phys.* **10**, 421–425 (2014).

27. L. J. Li, E. C. T. O'Farrell, K. P. Loh, G. Eda, B. Özyilmaz, A. H. Castro Neto, Controlling many-body states by the electric-field effect in a two-dimensional material. *Nature* **529**, 185–189 (2016).

28. O. Moulding, T. Muramatsu, C. J. Sayers, E. Da Como, S. Friedemann, Suppression of charge-density-wave order in $TiSe_2$ studied with high-pressure magnetoresistance. *Electron. Struct.* **4**, 035001 (2022).

29. M. Holt, P. Zschack, H. Hong, M. Y. Chou, T.-C. Chiang, X-ray studies of phonon softening in $TiSe_2$. *Phys. Rev. Lett.* **86**, 3799 (2001).

30. P. Knowles, B. Yang, T. Muramatsu, O. Moulding, J. Buhot, C. J. Sayers, E. Da Como, S. Friedemann, Fermi surface reconstruction and electron dynamics at the charge-density-wave transition in $TiSe_2$. *Phys. Rev. Lett.* **124**, 167602 (2020).

31. A. Kogar, G. A. de la Pena, S. Lee, Y. Fang, S. X.-L. Sun, D. B. Lioi, G. Karapetrov, K. D. Finkelstein, J. P. C. Ruff, P. Abbamonte, S. Rosenkranz, Observation of a charge density wave incommensuration near the superconducting dome in Cu_xTiSe_2. *Phys. Rev. Lett.* **118**, 027002 (2017).

32. H. Saqib, S. Rahman, Y. Zhao, C. Cazorla, D. Errandonea, R. Susilo, Y. Zhuang, Y. Huang, B. Chen, N. Dai, Evolution of structural and electronic properties of $TiSe_2$ under high pressure. *J. Phys. Chem. Lett.* **12**, 9859–9867 (2021).

33. W. Xia, J. Wu, Z. Li, J. Yuan, C. An, X. Wang, N. Yu, Z. Zou, G. Liu, C. Zhou, J. Feng, L. Zhang, Z. Dong, B. Chen, Z. Yang, Z. Yu, H. Chen, Y. Guo, Pressure-induced superconductivity reentrant in transition metal dichalcogenide $TiSe_2$. arXiv:2202.06244 [cond-mat.supr-con] (13 February 2022).

34. S. Lee, T. B. Park, J. Kim, S.-G. Jung, W. K. Seong, N. Hur, Y. Luo, D. Y. Kim, and T. Park, Tuning the charge density wave quantum critical point and the appearance of superconductivity in $TiSe_2$. *Phys. Rev. Res.* **3**, 033097 (2021).

35. I. M. Lifshitz, Anomalies of electron characteristics of a metal in the high pressure region. *Sov. Phys. JETP* **11**, 1130–1135 (1960).

36. C. Collignon, S. Badoux, S. A. A. Afshar, B. Michon, F. Laliberte, O. Cyr-Choiniere, J.-S. Zhou, S. Licciardello, S. Wiedmann, N. Doiron-Leyraud, L. Taillefer, Fermi-surface transformation across the pseudogap critical point of the cuprate superconductor $La_{1.6-x}Nd_{0.4}Sr_xCuO_4$. *Phys. Rev. B* **95**, 224517 (2017).

37. T. Hanaguri, K. Iwaya, Y. Kohsaka, T. Machida, T. Watashige, S. Kasahara, T. Shibauchi, Y. Matsuda, Two distinct superconducting pairing states divided by the nematic end point in $FeSe_{1-x}S_x$. *Sci. Adv.* **4**, eaar6419 (2018).

38. C. A. Marques, L. C. Rhodes, I. Benedicic, M. Naritsuka, A. B. Naden, Z. Li, A. C. Komarek, A. P. Mackenzie, P. Wahl, Atomic-scale imaging of emergent order at a magnetic fieldinduced Lifshitz transition. *Sci. Adv.* **8**, eabo7757 (2022).

39. S. Beaulieu, S. Dong, N. Tancogne-Dejean, M. Dendzik, T. Pincelli, J. Maklar, R. P. Xian, M. A. Sentef, M. Wolf, A. Rubio, L. Rettig, R. Ernstorfer, Ultrafast dynamical Lifshitz transition. *Sci. Adv.* **7**, eabd9275 (2021).

40. C. Liu, T. Kondo, R. M. Fernandes, A. D. Palczewski, E. D. Mun, N. Ni, A. N. Thaler, A. Bostwick, E. Rotenberg, J. Schmalian, S. L. Bud'ko, P. C. Canfield, A. Kaminski, Evidence for a Lifshitz transition in electron-doped iron arsenic superconductors at the onset of superconductivity. *Nat. Phys.* **6**, 419–423 (2010).

41. E. A. Yelland, J. M. Barraclough, W. Wang, K. V. Kamenev, A. D. Huxley, Highfield superconductivity at an electronic topological transition in URhGe. *Nat. Phys.* **7**, 890–894 (2011).

42. M. Monteverde, J. Lorenzana, P. Monceau, M. Nunez-Regueiro, Quantum critical point and superconducting dome in the pressure phase diagram of *o*-TaS_3. *Phys. Rev. B* **88**, 180504(R) (2013).

43. J. M. Park, Y. Cao, K. Watanabe, T. Taniguchi, P. Jarillo-Herrero, Tunable strongly coupled superconductivity in magic-angle twisted trilayer graphene. *Nature* **590**, 249–255 (2021).

44. J. van Wezel, P. Nahai-Williamson, and S. S. Saxena, Exciton-phonon interactions and superconductivity bordering charge order in $TiSe_2$. *Phys. Rev. B* **83**, 024502 (2011).

45. A. H. Wilson, The electrical conductivity of the transition metals. *Proc. R. Soc. Lond. A Math. Phys. Sci.* **167**, 580–593 (1938).

46. M. Gurvitch, Universal disorder-induced transition in the resistivity behavior of strongly coupled metals. *Phys. Rev. Lett.* **56**, 647 (1986).

47. S. Raymond, J. Bouchet, G. H. Lander, M. Le Tacon, G. Garbarino, M. Hoesch, J.-P. Rueff, M. Krisch, J. C. Lashley, R. K. Schulze, R. C. Albers, Understanding the complex phase diagram of uranium: The role of electron-phonon coupling. *Phys. Rev. Lett.* **107**, 136401 (2011).

48. T. Gruner, D. Jang, Z. Huesges, R. Cardoso-Gil, G. H. Fecher, M. M. Koza, O. Stockert, A. P. Mackenzie, M. Brando, C. Geibel, Charge density wave quantum critical point with strong enhancement of superconductivity. *Nat. Phys.* **13**, 967–972 (2017).

49. H. Shishido, R. Settai, H. Harima, Y. Onuki, A drastic change of the fermi surface at a critical pressure in $CeRhIn_5$: dHvA study under pressure. *J. Physical Soc. Jpn.* **74**, 1103–1106 (2005).

50. B. J. Ramshaw, S. E. Sebastian, R. D. McDonald, J. Day, B. S. Tan, Z. Zhu, J. B. Betts, R. Liang, D. A. Bonn, W. N. Hardy, N. Harrison, Quasiparticle mass enhancement approaching optimal doping in a high-T_c superconductor. *Science* **348**, 317–320 (2015).

51. K. Semeniuk, H. Chang, J. Baglo, S. Friedemann, S. W. Tozer, W. A. Coniglio, M. B. Gamża, P. Reiss, P. Alireza, I. Leermakers, A. M. Collam, A. D. Grockowiak, F. M. Grosche, Truncated mass divergence in a Mott metal. *Proc. Natl. Acad. Sci. U.S.A.* **120**, e2301456120 (2023).

52. M. D. Watson, O. J. Clark, F. Mazzola, I. Markovic, V. Sunko, T. K. Kim, K. Rossnagel, P. D. C. King, Orbital- and k_z-selective hybridization of Se 4*p* and Ti 3*d* states in the charge density wave phase of $TiSe_2$. *Phys. Rev. Lett.* **122**, 076404 (2019).

53. R. A. Craven, F. J. Di Salvo, F. S. L. Hsu, Mechanisms for the 200 K transition in $TiSe_2$: A measurement of the specific heat. *Solid State Commun.* **25**, 39–42 (1978).

54. P. Blaha, K. Schwarz, G. Madsen, D. Kvasnicka, J. Luitz, *WIEN2k* (19th ed., 2019).

55. J. Singleton, Studies of quasi-two-dimensional organic conductors based on BEDT-TTF using high magnetic fields. *Rep. Prog. Phys.* **63**, 1111–1207 (2000).

56. R. A. Jishi, H. M. Alyahyaei, Electronic structure of superconducting copper intercalated transition metal dichalcogenides: First-principles calculations. *Phys. Rev. B* **78**, 144516 (2008).

57. G. Wu, H. X. Yang, L. Zhao, X. G. Luo, T. Wu, G. Y. Wang, X. H. Chen, Transport properties of single-crystalline Cu_xTiSe_2 ($0.015 \leq x \leq 0.110$). *Phys. Rev. B* **76**, 024513 (2007).

58. D. Qian, D. Hsieh, L. Wray, E. Morosan, N. L. Wang, Y. Xia, R. J. Cava, M. Z. Hasan, Emergence of Fermi pockets in a new excitonic charge-density-wave melted superconductor. *Phys. Rev. Lett.* **98**, 117007 (2007).

59. J. F. Zhao, H. W. Ou, G. Wu, B. P. Xie, Y. Zhang, D. W. Shen, J. Wei, L. X. Yang, J. K. Dong, M. Arita, H. Namatame, M. Taniguchi, X. H. Chen, D. L. Feng, Evolution of the evolution of the electronic structure of 1*T*−Cu_xTiSe_2. *Phys. Rev. Lett.* **99**, 146401 (2007).

60. S. Kitou, A. Nakano, S. Kobayashi, K. Sugawara, N. Katayama, N. Maejima, A. Machida, T. Watanuki, K. Ichimura, S. Tanda, T. Nakamura, H. Sawa, Effect of Cu intercalation and pressure on excitonic interaction in 1*T*−$TiSe_2$. *Phys. Rev. B* **99**, 104109 (2019).

61. C. Bourbonnais, A. Sedeki, Superconductivity and antiferromagnetism as interfering orders in organic conductors. *C. R. Physique* **12**, 532–541 (2011).

62. K. Yamaji, Semimetallic SDW state in quasi one-dimensional conductors. *J. Physical Soc. Jpn.* **51**, 2787–2797 (1982).

63. S. Klotz, J.-C. Chervin, P. Munsch, G. Le Marchand, Hydrostatic limits of 11 pressure transmitting media. *J. Phys. D Appl. Phys.* **42**, 075413 (2009).

64. J. P. Perdew, K. Burke, and M. Ernzerhof, Generalized gradient approximation made simple. *Phys. Rev. Lett.* **77**, 3865 (1996).

65. D. J. Rahn, S. Hellmann, M. Kalläne, C. Sohrt, T. K. Kim, L. Kipp, K. Rossnagel, Gaps and kinks in the electronic structure of the superconductor 2*H*-$NbSe_2$ from angle-resolved photoemission at 1 K. *Phys. Rev. B* **85**, 224532 (2012).

66. T. Antonelli, W. Rahim, M. D. Watson, A. Rajan, O. J. Clark, A. Danilenko, K. Underwood, I. Markovic, E. Abarca-Morales, S. R. Kavanagh, P. Fevre, F. Bertran, K. Rossnagel, D. O. Scanlon, P. D. C. King, Orbital-selective band hybridisation at the charge density wave transition in monolayer $TiTe_2$. *NPJ Quantum Mater.* **7**, 98 (2022).

67. C. Monney, G. Monney, P. Aebi, H. Beck, Electron–hole instability in 1*T*-$TiSe_2$. *New J. Phys.* **14**, 075026 (2012).

68. C. J. Sayers, L. S. Farrar, S. J. Bending, M. Cattelan, A. J. H. Jones, N. A. Fox, G. Kociok-Köhn, K. Koshmak, J. Laverock, L. Pasquali, E. Da Como, Correlation between crystal purity and the charge density wave in $1T-VSe_2$. *Phys. Rev. Mater.* **4**, 025002 (2020).

69. B. Hildebrand, C. Didiot, A. M. Novello, G. Monney, A. Scarfato, A. Ubaldini, H. Berger, D. R. Bowler, C. Renner, P. Aebi, Doping nature of native defects in $1T-TiSe_2$. *Phys. Rev. Lett.* **112**, 197001 (2014).

70. M. S. Torikachvili, S. K. Kim, E. Colombier, S. L. Bud'ko, P. C. Canfield, Solidification and loss of hydrostaticity in liquid media used for pressure measurements. *Rev. Sci. Instrum.* **86**, 123904 (2015).
